# Potency of Omadacycline against Mycobacteroides abscessus Clinical Isolates *In Vitro* and in a Mouse Model of Pulmonary Infection

**DOI:** 10.1128/AAC.01704-21

**Published:** 2022-01-18

**Authors:** Danielle A. Nicklas, Emily C. Maggioncalda, Elizabeth Story-Roller, Benjamin Eichelman, Chavis Tabor, Alisa W. Serio, Tiffany R. Keepers, Surya Chitra, Gyanu Lamichhane

**Affiliations:** a Division of Infectious Diseases, Department of Medicine, School of Medicine, Johns Hopkins University School of Medicine, Baltimore, Maryland, USA; b Paratek Pharmaceuticals, Inc., King of Prussia, Pennsylvania, USA

**Keywords:** omadacycline, *Mycobacterium abscessus*, pulmonary infection

## Abstract

The incidence of nontuberculous mycobacterial diseases in the United States is rising and has surpassed that of tuberculosis. Most notable among the nontuberculous mycobacteria is Mycobacteroides abscessus, an emerging environmental opportunistic pathogen capable of causing chronic infections. M. abscessus disease is difficult to treat, and the current treatment recommendations include repurposed antibiotics, several of which are associated with undesirable side effects. In this study, we have evaluated the activity of omadacycline, a new tetracycline derivative, against M. abscessus using *in vitro* and *in vivo* approaches. Omadacycline exhibited an MIC_90_ of 0.5 µg/mL against a panel of 32 contemporary M. abscessus clinical isolates, several of which were resistant to antibiotics that are commonly used for treatment of M. abscessus disease. Omadacycline combined with clarithromycin, azithromycin, cefdinir, rifabutin, or linezolid also exhibited synergism against several M. abscessus strains and did not exhibit antagonism when combined with an additional nine antibiotics also commonly considered to treat M. abscessus disease. Concentration-dependent activity of omadacycline was observed in time-kill assessments. Efficacy of omadacycline was evaluated in a mouse model of lung infection against four M. abscessus strains. A dose equivalent to the 300-mg standard oral human dose was used. Compared to the untreated control group, within 4 weeks of treatment, 1 to 3 log_10_ fewer M. abscessus CFU were observed in the lungs of mice treated with omadacycline. Treatment outcome was biphasic, with bactericidal activity observed after the first 2 weeks of treatment against all four M. abscessus strains.

## INTRODUCTION

Mycobacteroides abscessus (formerly Mycobacterium abscessus) (1) is an environmental nontuberculous mycobacterium (NTM) and a causative pathogen of pulmonary and soft tissue infections, among others. This organism has been described as a “clinical and antibiotic nightmare” (2, 3), as it demonstrates intrinsic resistance to a wide range of antibiotic classes (4, 5). As only a few oral antibiotics show activity against M. abscessus, the current treatment regimen, which requires regular and frequent administration of intravenous drugs is a logistical challenge for patients. Acquired resistance to important antibiotic classes, particularly macrolides and aminoglycosides, has further limited therapeutic options. Therefore, M. abscessus disease treatment is challenging, and there are currently no FDA approved treatment agents or regimens for this indication. Current treatment options include repurposed antibiotics and regimens developed on the basis of empirical evidence and consensus from experts (6–10). Cure rates using antibiotics alone are as low as 25 to 40% (11, 12).

M. abscessus is one of the most frequently recovered NTM from patients, causing ∼10% of all NTM pulmonary infections (13). It was the second most common NTM group identified in a large registry of bronchiectasis patients in the United States (14). Furthermore, a steady increase in the incidence of M. abscessus was observed in U.K. clinics from 2000 to 2013 (15). A recent study of a cystic fibrosis patient cohort in the United States examined 341 NTM strains and reported that M. abscessus comprised the majority of the isolates (16). These results suggest that the incidence of M. abscessus is increasing, and so too will the need for new treatments that are effective options against this emergent disease. Additionally, many patients with chronic M. abscessus disease that are refractory to prescribed treatment are likely to harbor strains resistant to the antibiotics that comprise their treatment regimen (17). An ideal new treatment would also be effective against these antibiotic-resistant strains.

Omadacycline is an aminomethylcycline, which is a semisynthetic derivative of the tetracycline class (18). It is a broad-spectrum antibiotic (19) that received FDA approval in 2018 for treatment of acute bacterial skin and skin structure infections and community-acquired bacterial pneumonia, and it is available in intravenous and oral formulations (20). Tigecycline is another tetracycline that is included in the recommendation for treating M. abscessus disease and often used in combination with other antibiotics (8, 10). Tigecycline and omadacycline exhibit similar *in vitro* activity against a range of clinical M. abscessus isolates (21–23). Additional studies that have demonstrated potent *in vitro* activity of omadacycline against NTM, including M. abscessus (24, 25), have generated further interest in this antibiotic for treating M. abscessus disease. Some advantages of omadacycline compared to tigecycline include (i) that omadacycline is available in both intravenous and oral formulations, making administration simpler for patients and allowing for oral therapy, (ii) that omadacycline has an elevated and sustained concentration in epithelial lining fluid, alveolar cells, and plasma, as well as improved pulmonary pharmacokinetics (PK) compared to those of tigecycline (26), and (iii) that omadacycline may have better tolerability than tigecycline (25). In addition, in a recent preliminary, real-world multicenter study, clinical success has been demonstrated with regimens containing omadacycline to treat M. abscessus infections in the majority of patients (27).

In clinical trials for treatment of acute bacterial infections of the skin and skin structures and of community-acquired bacterial pneumonia, omadacycline was found to be noninferior and tolerable compared to standard-of-care treatments (28–31). In preclinical studies, omadacycline demonstrated dose-dependent activity against Streptococcus pneumoniae in a murine pneumonia model (32) and against Staphylococcus aureus in neutropenic murine pneumonia and thigh infection models (33, 34). Based on the demonstrated efficacy and tolerability in treating pneumonia in clinical trials, efficacy in murine pneumonia models, and *in vitro* activity against M. abscessus, we asked whether omadacycline has efficacy in treating M. abscessus lung disease. Here, using a collection of distinct contemporary M. abscessus clinical isolates, we evaluated the activity of omadacycline *in vitro* and *in vivo* and compared it to the standard of care where applicable. Our studies included determination of MIC, time-kill analysis, *in vitro* activity in combination with other antibiotics, and efficacy in a mouse model of pulmonary M. abscessus infection (35).

## RESULTS

### MICs of omadacycline against clinical isolates of M. abscessus.

We initiated this study by determining the MICs of omadacycline and other antibiotics that have shown *in vitro* activity in prior studies, some of which are included in the current treatment recommendations for M. abscessus infection in humans (6–8, 10). These include tigecycline, amikacin, clarithromycin, azithromycin, linezolid, moxifloxacin, rifabutin, vancomycin, teicoplanin, doripenem, imipenem, cefoxitin, cefdinir, ceftazidime, and amoxicillin, and those that have shown promise in recent studies, such as clofazimine and bedaquiline (17, 36–45). These antibiotics represent a broad spectrum of antibiotic classes, including tetracycline, aminoglycoside, macrolide, fluoroquinolone, rifamycin, glycopeptide, β-lactam, phenazine, and diarylquinoline classes.

Thirty-one independent M. abscessus clinical strains were included. The majority of strains (29 of 31) were isolated from cystic fibrosis and bronchiectasis patients (17), and two strains were from gastrointestinal infections (46). In addition, American Type Culture Collection (ATCC) 19977, a strain that has been historically designated the reference M. abscessus strain and is widely used by laboratories studying this organism (47), was also included. Of the 32 M. abscessus strains, 17 belonged to M. abscessus subsp. *abscessus* and 10 were M. abscessus subsp. *massiliense.* Subspecies determination for the remaining 5 strains has not been completed. Subspecies determination was undertaken using whole-genome sequencing as described previously (48). For MIC determination, in addition to the cation-adjusted Mueller-Hinton broth (CAMHB) that is recommended by the Clinical and Laboratory Standards Institute (CLSI) (49), we also used Middlebrook 7H9 broth to assess if there were any distinct differences in MIC values obtained with the different medium types.

The MICs of each drug were determined in duplicate against each strain in CAMHB (Table 1) and Middlebrook 7H9 broth (see Table S1 in the supplemental material); if two different MIC values were obtained, an average MIC value was calculated (e.g., an independent MIC of 0.5 and 1 µg/mL were averaged to 0.75 µg/mL). The omadacycline MIC values in CAMHB ranged from 0.06 to 1 µg/mL, with MIC_50_ and MIC_90_ values of 0.25 µg/mL and 0.5 µg/mL, respectively, against the 32 tested strains. The median omadacycline MIC value was 0.25 µg/mL for both M. abscessus subsp. *abscessus* and M. abscessus subsp. *massiliense.* In Middlebrook 7H9 broth, omadacycline MIC values ranged from 0.25 µg/mL to 2 µg/mL, with MIC_50_ and MIC_90_ values of 0.5 µg/mL and 1.0 µg/mL, respectively. The median omadacycline MIC values were 0.5 and 0.75 µg/mL for M. abscessus subsp. *abscessus* and M. abscessus subsp. *massiliense*, respectively.

**TABLE 1:** MICs of omadacycline and select antibiotics against 31 M. abscessus clinical isolates and reference strain ATCC 19977 in CAMHB

Drug^*b*^	MIC (µg/mL) against isolate of^*a*^:																																MIC range (µg/mL)	MIC_50_ (µg/mL)	MIC_90_ (µg/mL)
	M. abscessus subsp. *abscessus*													M. abscessus subsp. *massiliense*									M. abscessus subsp. ND												
	ATCC 19977	M9501	M9503	M9507	M9513	M9522	M9525	M9526	M9527	M9528	M9529	M9530	M9531	M9502	M9504	M9505	M9509	M9510	M9514	M9515	M9517	M9521	M9508	M9518	M9519	M9523	M9524	M9533	M9534	M9551	M9563	M9565			
OMC	0.375	0.25	0.125	0.5	0.25	0.25	0.375	0.25	0.25	0.5	1	0.25	0.25	0.375	0.25	0.188	0.06	0.5	0.375	1	0.188	0.25	0.125	0.125	0.125	0.06	0.75	0.25	0.25	0.5	0.25	0.188	0.06 to 1	0.25	0.5
TGC	0.125	0.25	0.06	0.25	0.093	0.188	0.188	0.125	0.25	0.125	1	0.125	0.25	0.375	0.093	0.093	≤0.06	0.25	0.188	0.5	0.188	0.093	0.188	0.125	0.125	≤0.06	0.375	0.125	0.125	0.25	0.25	0.093	0.06 to 1	0.125	0.375
AMK	16	16	16	**>256**	8	16	**>256**	**>256**	**>256**	**>256**	**>256**	16	16	16	16	12	8	8	12	8	16	16	8	8	8	16	12	16	16	16	16	12	8 to >256	16	>256
CLR	1	≤0.06	0.25	4	0.75	0.5	**8**	2	4	4	1	3	4	≤0.06	≤0.06	≤0.06	≤0.06	0.125	≤0.06	≤0.06	≤0.06	≤0.06	0.375	0.125	0.25	≤0.06	0.125	0.5	≤0.06	≤0.06	2	≤0.06	0.06 to 8	0.125	4
AZM	16	0.5	16	32	24	8	64	64	48	64	32	64	64	1	0.5	0.375	0.25	1	0.75	1	1.5	0.5	8	4	8	0.25	4	16	1	1	16	1	0.25 to 64	4	64
IMI	24	16	24	24	24	24	24	**32**	24	24	**256**	**48**	24	16	24	16	24	**64**	16	**32**	16	24	16	**48**	24	**64**	24	24	24	**32**	24	24	16 to 256	24	48
DOR	16	24	16	24	24	24	24	16	32	32	32	32	32	32	32	16	16	64	48	64	32	16	32	64	32	64	24	32	32	32	64	32	16 to 64	32	64
FOX	16	32	16	32	16	16	24	16	32	32	32	32	32	32	32	16	16	32	16	48	16	16	32	32	32	32	32	32	32	32	32	32	16 to 48	32	32
CDR	64	64	32	96	64	32	64	32	96	128	64	64	64	32	48	16	8	64	16	64	32	16	192	256	192	128	48	128	128	64	128	64	8 to 256	64	128
CAZ	>256	>256	>256	>256	>256	>256	>256	>256	>256	>256	>256	>256	>256	>256	>256	>256	>256	>256	>256	>256	>256	>256	>256	>256	>256	>256	>256	>256	>256	>256	>256	>256	>256	>256	>256
AMX	>256	>256	>256	>256	>256	>256	>256	>256	>256	>256	>256	>256	>256	>256	>256	>256	>256	>256	>256	>256	>256	>256	>256	>256	>256	>256	>256	>256	>256	>256	>256	>256	>256	>256	>256
VAN	>256	>256	>256	>256	>256	>256	>256	>256	>256	>256	>256	>256	>256	>256	>256	>256	>256	>256	>256	>256	>256	>256	>256	>256	>256	128	>256	>256	>256	>256	>256	>256	>256	>256	>256
TEC	>256	>256	>256	>256	>256	>256	>256	>256	>256	>256	>256	>256	>256	>256	>256	>256	>256	>256	>256	>256	>256	>256	>256	>256	>256	>256	>256	>256	>256	>256	>256	>256	>256	>256	>256
LZD	**32**	**32**	**32**	**64**	16	**32**	**32**	16	**48**	**32**	**32**	**64**	**64**	**64**	16	16	8	16	**64**	**32**	24	16	16	8	12	3	**32**	16	**32**	**64**	**64**	**64**	3 to 64	32	64
CFZ	0.25	0.25	0.188	0.188	0.375	0.375	0.25	0.125	0.25	0.25	0.188	0.375	0.188	0.25	0.25	0.25	0.093	1	0.25	0.188	0.25	0.25	0.093	0.093	0.188	0.125	0.375	0.25	0.25	0.188	0.25	0.125	0.09 to 1	0.25	0.375
MOX	**16**	**16**	**16**	**32**	**16**	**32**	**16**	**16**	**64**	**32**	**64**	**8**	**16**	**32**	**8**	**8**	**8**	**32**	**32**	**64**	**8**	**8**	**6**	**8**	**8**	2	**16**	**8**	**8**	**16**	**16**	**6**	2 to 64	16	32
RFB	8	8	8	8	8	16	8	8	8	8	16	16	16	16	8	4	4	4	8	4	8	8	8	4	8	1.5	16	12	8	16	32	6	1.5 to 32	8	16
BDQ	<0.06	≤0.06	≤0.06	≤0.06	≤0.06	≤0.06	≤0.06	≤0.06	≤0.06	≤0.06	≤0.06	≤0.06	≤0.06	≤0.06	≤0.06	≤0.06	≤0.06	≤0.06	≤0.06	≤0.06	≤0.06	≤0.06	≤0.06	≤0.06	≤0.06	≤0.06	≤0.06	≤0.06	≤0.06	≤0.06	≤0.06	≤0.06	≤0.06	≤0.06	≤0.06

a MIC values are averages of two biological replicates. For agents with published CLSI breakpoints, resistant MIC values are denoted in bold and intermediate MIC values are denoted by underlines (49) (see Table S2 in the supplemental material). ND, M. abscessus isolates whose subspecies has not been determined.

b OMC, omadacycline; TGC, tigecycline; AMK, amikacin; CLR, clarithromycin; AZM, azithromycin; IMI, imipenem; DOR, doripenem; FOX, cefoxitin; CDR, cefdinir; CAZ, ceftazidime; AMX, amoxicillin; VAN, vancomycin; TEC, teicoplanin; LZD, linezolid; CFZ, clofazimine; MOX, moxifloxacin; RFB, rifabutin; BDQ, bedaquiline.

The MICs of certain antibiotics against M. abscessus differed between the two broths. MIC values of omadacycline, clarithromycin, azithromycin, cefoxitin, cefdinir, linezolid, clofazimine, and rifabutin consistently trended lower for a majority of M. abscessus strains in CAMHB broth compared to MIC values in Middlebrook 7H9 broth. For example, of the 32 isolates tested, 78% (25 of 32) had omadacycline MIC values ≥2-fold higher in Middlebrook 7H9 broth compared to those in CAMHB. While a 2-fold variation is typically within the standard acceptable variability of an MIC assay, this trend should be reported. Further, 31% (10 of 32) of isolates tested indeed had omadacycline MIC values ≥4-fold higher in Middlebrook 7H9 broth compared to those in CAMHB, which is considered meaningful. MICs of some other antibiotics were even more strikingly different; for example, 100% (32 of 32) of azithromycin MIC values were ≥2-fold higher in Middlebrook 7H9 broth compared to those in CAMHB, and of these, 75% (24 of 32) were ≥4-fold higher and 66% (21 of 32) were ≥8-fold higher. On the other hand, the MIC values of imipenem consistently trended higher in CAMHB broth compared to those in Middlebrook 7H9 broth, where MIC values were ≥2-fold for 68% (19 of 32) of the M. abscessus strains (note that only 9% [3 of 32] differed by ≥4-fold). These data highlight the need for the utilization of consistent and appropriate media for susceptibility testing, and therefore CAMHB was utilized for other *in vitro* testing in this study in accordance with CLSI guidelines.

### Omadacycline is active *in vitro* against M. abscessus clinical isolates that are resistant to currently used antibiotics.

Several strains included in this study were resistant to a range of antibiotics based on CLSI breakpoints (highlighted in Table 1). The collection of M. abscessus strains included in our study (*n* = 32) were randomly selected without prior knowledge of MIC values to omadacycline, and the MIC results showed that 100% were inhibited by ≤1 µg/mL of omadacycline when tested according to CLSI methodology, confirming its potent *in vitro* activity. The MIC of omadacycline was consistently within one dilution of its median MIC (0.25 µg/mL in CAMHB) against strains that displayed a high level of resistance to antibiotics that are frequently used to treat M. abscessus disease, such as amikacin, clarithromycin, azithromycin, cefoxitin, and imipenem. The MIC of omadacycline was also within one dilution of its median MIC against strains that were resistant to antibiotics less commonly used to treat M. abscessus disease, such as moxifloxacin, doripenem, and linezolid.

### Omadacycline in combination with clarithromycin, azithromycin, cefdinir, linezolid, or rifabutin exhibits synergy *in vitro* against a subset of clinical M. abscessus isolates.

**FIG 1:**
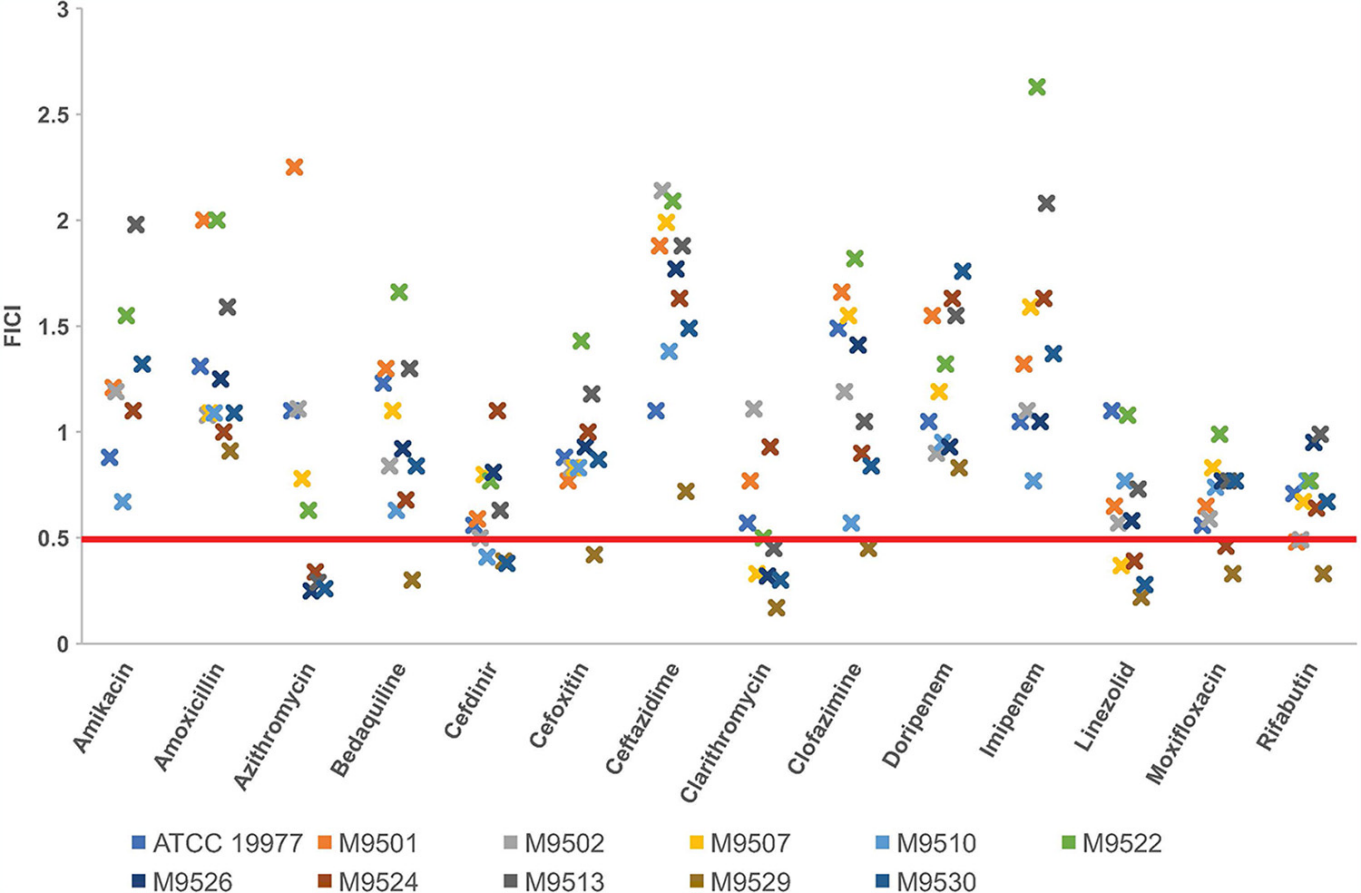
Combined activity of omadacycline and another antibiotic against 11 M. abscessus isolates. A fractional inhibitory concentration index (FICI) of ≤0.5 was interpreted as synergy, >0.5 to 4 as indifference, and >4 as antagonism, per the most stringent recommendation (51). The red line at a FICI of 0.5 demarcates synergy (at or below the line) from indifference (above the line). Omadacycline was synergistic with the antibiotic indicated for strains at or below the red line. Each ‘x’ mark denotes a single M. abscessus strain.

Using a checkerboard assay (50), we assessed if omadacycline acts with synergy, indifference, or antagonism when combined with antibiotics currently used to treat M. abscessus disease. For this assessment, omadacycline in combination with 14 different antibiotics from a wide range of classes were tested against 10 M. abscessus clinical isolates and the reference strain ATCC 19977 (Fig. 1). The most stringent interpretation of combined activity of the antibiotics (51) was used. Omadacycline in combination with clarithromycin exhibited synergy against 6 of the 11 (55%) strains tested, as indicated by the fractional inhibitory concentration index (FICI) of ≤0.5. Omadacycline also exhibited synergy in combination with azithromycin, cefdinir, or linezolid against four of the tested strains (36%), as well as with rifabutin against three strains (27%). Omadacycline in combination with amikacin, imipenem, doripenem, ceftazidime, or amoxicillin did not exhibit synergy against any of the 11 M. abscessus strains. We did not observe antagonistic activity of omadacycline when it was combined with any of the antibiotics tested. Strain-specific synergy trends were not observed (Fig. 1). We also determined that when omadacycline was combined with clarithromycin, azithromycin, cefdinir, or linezolid, the combination often reduced the MIC values of these antibiotics against M. abscessus strains from intermediate/resistant to susceptible (Table S3), according to CLSI interpretive criteria (Table S2). For example, the MIC of clarithromycin when assessed alone against isolate M9501 was 4.0 µg/mL (Table 1) and would be considered intermediate according to CLSI guidelines (Table S2). In combination with omadacycline, the MIC of clarithromycin was 0.5 µg/mL (Table S3), as this is the lowest concentration of clarithromycin at which growth of M9501 could not be observed, irrespective of omadacycline concentration (Table S4).

### Time-kill activity of omadacycline against M. abscessus.

**FIG 2:**
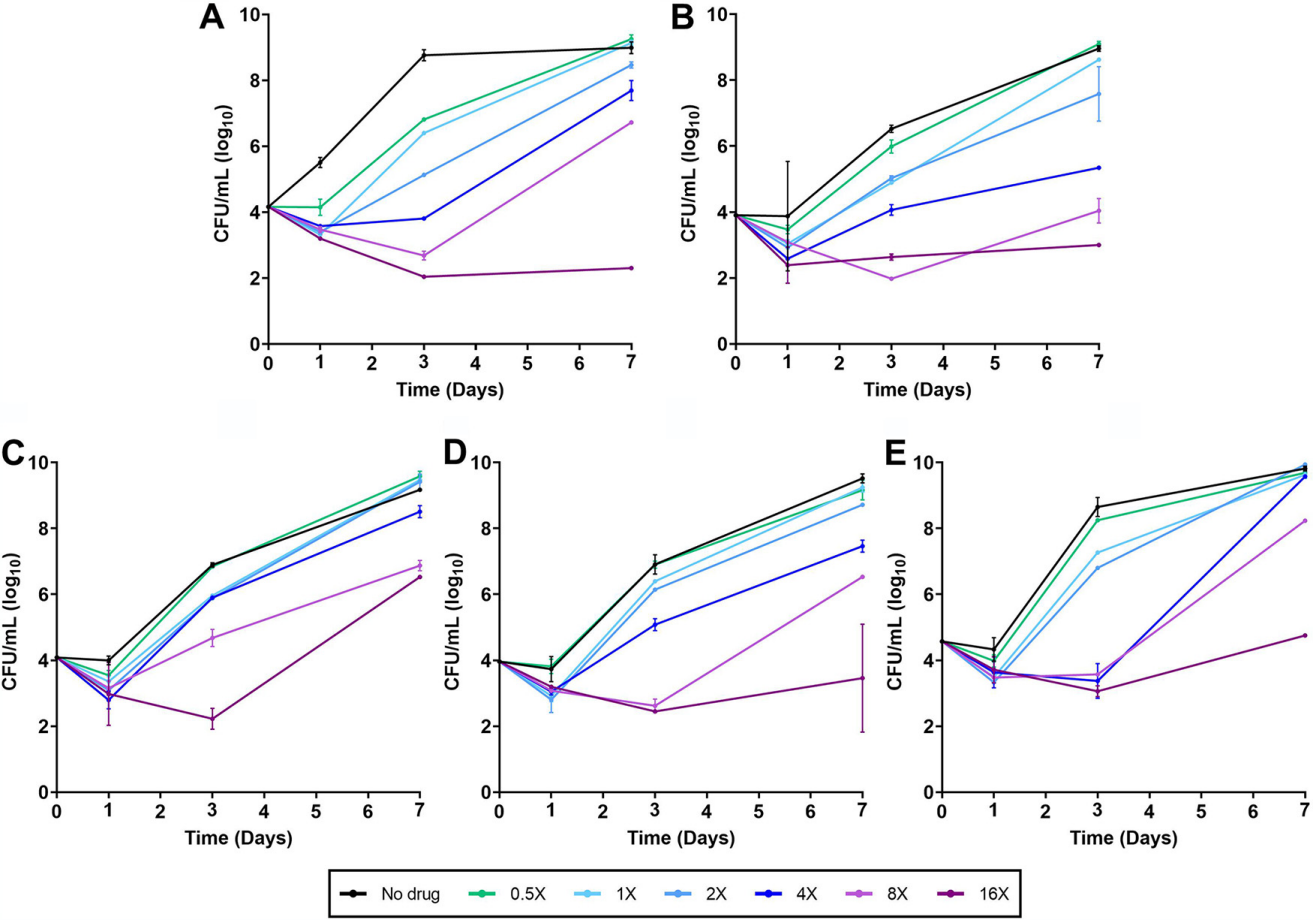
Time-kill activity of omadacycline against five M. abscessus isolates. M. abscessus strain ATCC 19977 (A), and clinical isolates M9510 (B), M9526 (C), M9529 (D), and M9530 (E) were exposed to omadacycline at 0.5×, 1×, 2×, 4×, 8×, and 16× MIC specific to each isolate and to no drug in cation-adjusted Mueller-Hinton broth (CAMHB). Surviving colonies were recovered on CAMHB agar in duplicate at 1, 3, and 7 days and enumerated (mean ± standard deviation [SD]).

MIC and FICI assays only provide one time point for the inhibitory activity of an antibiotic on bacterial growth. To generate insight into the activity of omadacycline over time, we undertook time-kill assessments against ATCC 19977 and four randomly selected M. abscessus clinical isolates. Each strain was exposed to a single dose of omadacycline at 0.5×, 1×, 2×, 4×, 8×, and 16× the MIC specific to each strain. As a control, each strain was also assessed in the absence of antibiotic. Omadacycline exhibited concentration-dependent activity, as demonstrated during the first 24 h of exposure, where omadacycline at 1× MIC and increasing fold concentrations above the MIC reduced the CFU levels of all isolates (Fig. 2). Time-dependent activity was also observed against all isolates, where further reductions in CFU occurred until 3 days when the isolates were exposed to 8× MIC or higher concentrations of omadacycline. However, at lower concentrations, M. abscessus strains were able to recover after 3 days of exposure and exhibited sustained growth. CFU levels at 3 and 7 days postexposure correlated inversely with the concentration of omadacycline.

**FIG 3:**
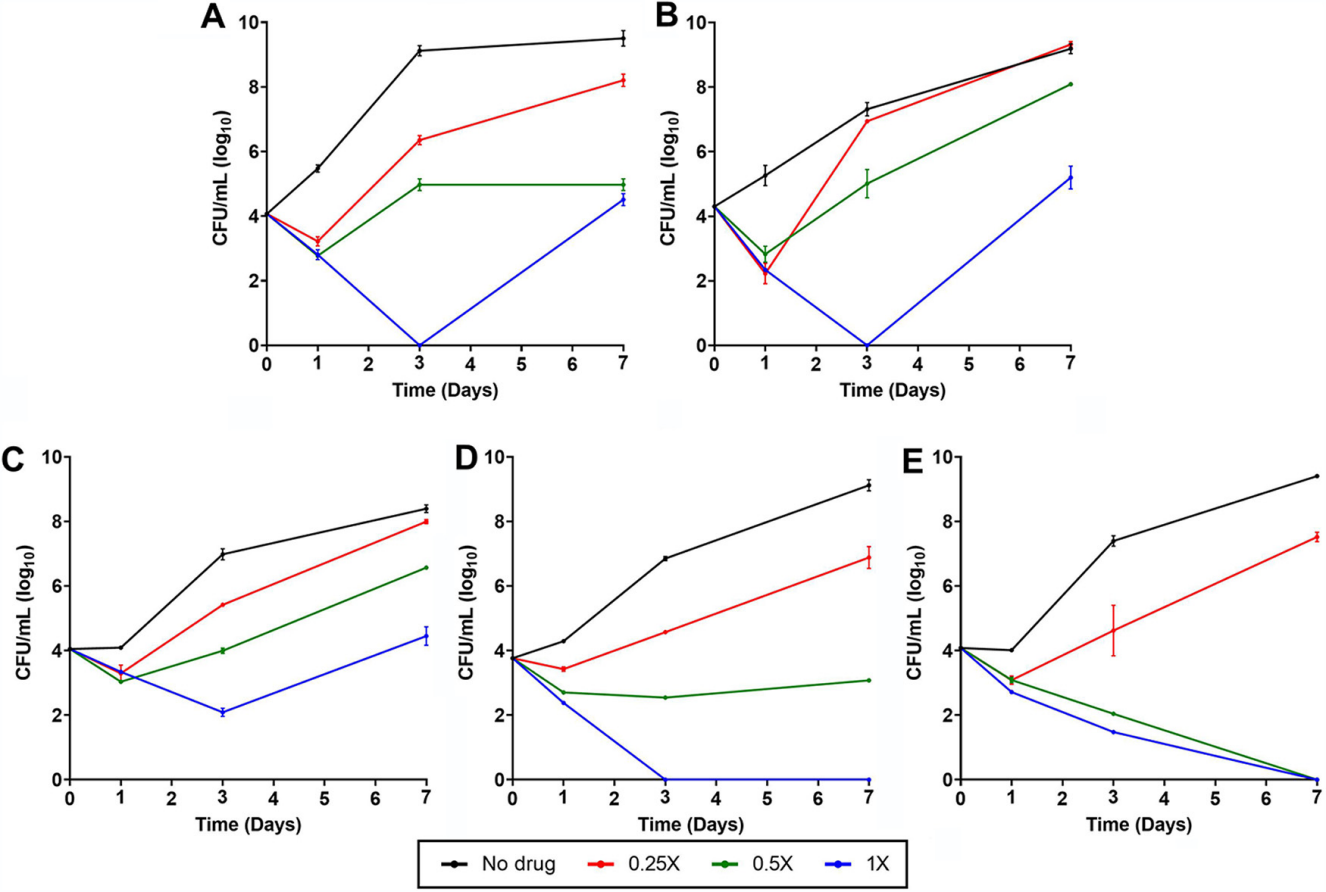
Time-kill activity of omadacycline and rifabutin against five M. abscessus isolates. M. abscessus strain ATCC 19977 (A) and clinical isolates M9510 (B), M9526 (C), M9529 (D), and M9530 (E) were exposed to a combination of omadacycline and rifabutin at 0.25×, 0.5×, and 1× MIC of each antibiotic specific to each strain and to no drug in CAMHB. Surviving colonies were recovered on CAMHB agar in duplicate at 1, 3, and 7 days and enumerated (mean ± SD). Additional time-kill curves against five other synergistic pairs are included in Fig. S1 and S5 in the supplemental material.

In addition to omadacycline alone, we also undertook time-kill analysis of omadacycline in combination with clarithromycin, azithromycin, cefdinir, linezolid, and rifabutin, as they exhibited synergy against some M. abscessus strains in checkerboard assays. Amikacin was also included, as it is one of the most frequently utilized antibiotics to treat M. abscessus disease. For antibiotic pairs, 0.25×, 0.5×, and 1× the respective MICs of omadacycline and each antibiotic were evaluated in combination against the same five M. abscessus isolates, as described above. Variable bactericidal activity among different clinical isolates for the same antibiotic combination was observed. For instance, when omadacycline and rifabutin were combined (Fig. 3), bactericidal activity was observed at 1× MIC of each antibiotic against the reference strain ATCC 19977 and M9510 for up to 3 days, but the growth of these isolates recovered thereafter. For M9529 and M9530, this same antibiotic combination at 1× MIC was bactericidal throughout and resulted in no detectable CFU at 7 days postexposure. Based on reduction in CFU counts, of the six antibiotic pairs evaluated, the combination of omadacycline and rifabutin exhibited the most potent time-kill activity profile against the greatest number of isolates. The combinations of omadacycline plus clarithromycin, azithromycin, cefdinir, linezolid, or amikacin produced reduction in CFU counts of most isolates during the first 24 h of exposure (see Fig. S1 to S5 in the supplemental material). Beyond 24 h, each drug produced variable changes in the CFU counts of the five isolates. As the objective of this assessment was to compare activities of combinations of omadacycline and a companion antibiotic at different concentrations over a defined duration against M. abscessus isolates, single-antibiotic comparators were not included. Therefore, the M. abscessus CFU changes reflect the net activities of the two-drug combinations specified in the assessments.

### Pharmacokinetics of omadacycline in C3HeB/FeJ and BALB/c mice.

To identify the omadacycline dose that best represents the human equivalent exposure or area under the plasma concentration versus time curve from time zero to 24 h after dosing (AUC_0–24_) of the human 300-mg oral dose (20), we determined pharmacokinetic (PK) parameters of omadacycline in C3HeB/FeJ and BALB/c mice. The human steady-state AUC_0–24_ for the 300-mg dose is 11.156 h · µg/mL for total drug and 8.92 h · µg/mL for free drug, based on 20% plasma protein binding in humans (Nuzyra prescribing information). The C3HeB/FeJ mouse strain PK parameters were determined because this mouse strain was utilized to evaluate omadacycline efficacy in an M. abscessus pulmonary infection model (35). PK parameters were also determined in the BALB/c mouse strain, as this strain has also been considered for studying M. abscessus infection (46, 52). Additionally, the BALB/c strain is used in *in vivo* efficacy studies against related nontuberculous mycobacteria such as Mycobacterium avium (53), Mycobacterium ulcerans (54), and Mycobacterium tuberculosis (55, 56). Furthermore, BALB/c mouse plasma is commercially available to facilitate determination of the free and bound fraction of omadacycline. Therefore, the BALB/c data set is not only important for potential future work but also aids in determination of free AUC_0–24_ for C3HeB/FeJ mice in the absence of commercially available C3HeB/FeJ mouse plasma.

**FIG 4:**
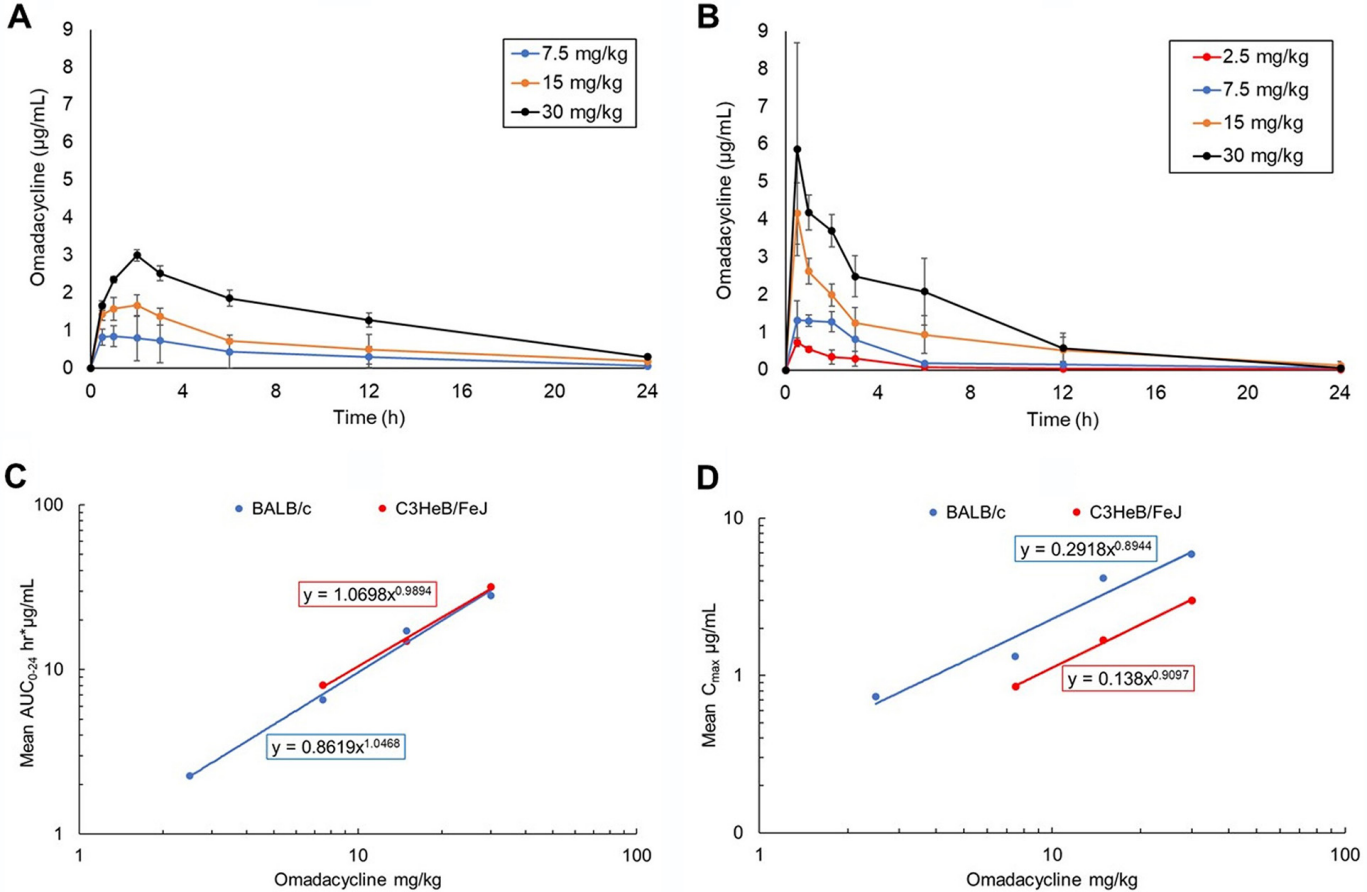
Omadacycline pharmacokinetic (PK) parameters in C3HeB/FeJ and BALB/c mice. Mean (±SD) omadacycline plasma concentration versus scheduled time points in C3HeBFeJ mice (A) and BALB/c mice (B). Dose linearity of the area under the plasma concentration versus time curve from time zero to 24 h after dosing (AUC_0–24_) (D)and *C*_max_ (D) in BALB/c and C3HeB/FeJ mice.

Omadacycline pharmacokinetic (PK) parameters were determined in C3HeB/FeJ mice by taking plasma samples at 0.5, 1, 2, 3, 6, 12, and 24 h after subcutaneous injection of omadacycline at 7.5, 15, or 30 mg/kg. Omadacycline PK parameters were also determined in BALB/c mice by taking plasma samples at 0.25, 0.5, 1, 2, 6, 12, and 24 h after intraperitoneal injection of omadacycline at 2.5, 7.5, 15, or 30 mg/kg. Plasma concentrations of omadacycline versus time were plotted (Fig. 4A and B) to determine PK parameters for C3HeB/FeJ mice (Table 2) and for BALB/c mice (Table 3). For C3HeB/FeJ mice, both AUC_0–24_ and maximum observed plasma concentration (*C*_max_) were dose linear within the dose range of 7.5 to 30 mg/kg (Fig. 4C and D). The AUC_0–24_ is numerically dose proportional (slope = 0.99), and the *C*_max_ is numerically less than dose proportional (slope = 0.91; <1.0). For BALB/c mice, both AUC_0–24_ and *C*_max_ were also dose linear (Fig. 4C and D). The AUC_0–24_ is numerically dose proportional (slope = 1.05), and the *C*_max_ is numerically less than dose proportional (slope = 0.89; <1.0). These data show that omadacycline AUC_0–24_ and *C*_max_, while similar in these two mouse strains at the doses tested, are not identical, with differences in PK parameters attributable to the different routes of administration. Specifically, intraperitoneal administration typically yields higher *C*_max_ values than does subcutaneous injection (57).

**TABLE 2:** Omadacycline PK parameters in C3HeB/FeJ mice dosed via subcutaneous injection

Omadacycline dose (mg/kg)	AUC_0–24_ (h · µg/mL)^*a*^		*C*_max_ (µg/mL)^*b*^	*t*_1/2_ (h)^*c*^
	Total	Free		
7.5	8.07	4.53	0.85	5.85
15.0	14.77	8.30	1.67	8.86
30.0	31.81	17.87	3.00	6.81

a AUC_0–24_, area under the plasma concentration versus time curve (AUC) from time zero to 24 h after dosing, calculated using the linear trapezoidal linear interpolation method. Free AUC_0-24h_ was determined based on 33.9% plasma protein binding in BALB/c mice.

b *C*_max_, maximum observed plasma concentration.

c *t*_1/2_, plasma terminal elimination half-life, calculated as *t*_1/2_=ln(2)/λz, where λz is the terminal elimination rate constant calculated by linear regression of the terminal portion of the natural log of plasma concentration versus time curve.

**TABLE 3:** Omadacycline PK parameters in BALB/c mice dosed via intraperitoneal injection

Omadacycline dose (mg/kg)	AUC_0–24_ (h · µg/mL)^*a*^		*C*_max_ (µg/mL)^*b*^	*t*_1/2_ (h)^*c*^
	Total	Free^*a*^		
2.5	2.26	1.49	0.73	4.21
7.5	6.51	4.30	1.32	9.53
15.0	17.18	11.36	4.16	6.31
30.0	28.13	18.59	5.87	3.33

a Free AUC_0–24_ was determined based on 33.9% plasma protein binding in BALB/c mice. AUC_0–24_, area under the plasma concentration versus time curve (AUC) from time zero to 24 h after dosing, calculated using the linear trapezoidal linear interpolation method.

b *C*_max_, maximum observed plasma concentration.

c *t*_1/2_, plasma terminal elimination half-life, calculated as *t*_1/2_ = ln(2)/λz, where λz is the terminal elimination rate constant calculated by linear regression of the terminal portion of the natural log of plasma concentration versus time curve.

Free AUC_0–24_ values were determined using an average mouse plasma protein binding of 33.9%, determined in BALB/c mice via equilibrium dialysis (data not shown). Linear regression analysis of the dose versus the C3HeB/FeJ mouse free AUC_0–24_ was performed and determined that an omadacycline subcutaneous dose of 15 mg/kg would result in a mouse free AUC_0–24_ of 9.2 h · µg/mL, which best represents the human free AUC_0–24_ of 8.92 h · µg/mL. It is of note that omadacycline lacks oral bioavailability in rodents (Paratek Pharmaceuticals, personal communication) and thus extrapolation of the AUC obtained via subcutaneous administration in the mouse to the AUC obtained via oral administration in the human was required. Omadacycline concentrations in plasma from infected and uninfected C3HeB/FeJ mice were also measured. Plasma samples from infected and uninfected mice were compared and showed no significant difference in omadacycline concentrations, indicating that M. abscessus infection had no effect on omadacycline plasma levels in mice (data not shown).

### Efficacy of omadacycline against M. abscessus pulmonary infection in mice.

The efficacy of omadacycline was evaluated against four independent M. abscessus isolates in a mouse model of pulmonary M. abscessus infection (35). These strains were ATCC 19977 and recent pulmonary clinical isolates M9501, M9529, and M9530. The four isolates have a range of MIC values for antibiotics most frequently used to treat M. abscessus infections, such as amikacin (16 to >256 μg/mL), clarithromycin (≤0.06 to 3 μg/mL), and imipenem (16 to 256 μg/mL) and were thus chosen to represent different phenotypes that may be encountered in the clinic (Table 1). For example, the clinical isolate M9501 is susceptible to most antibiotics, M9529 is resistant to both amikacin and imipenem, and M9530 is resistant to imipenem. All three clinical isolates and ATCC 19977 are resistant to linezolid and moxifloxacin. Omadacycline MIC values ranged from 0.25 to 1 μg/mL against the four isolates tested.

**FIG 5:**
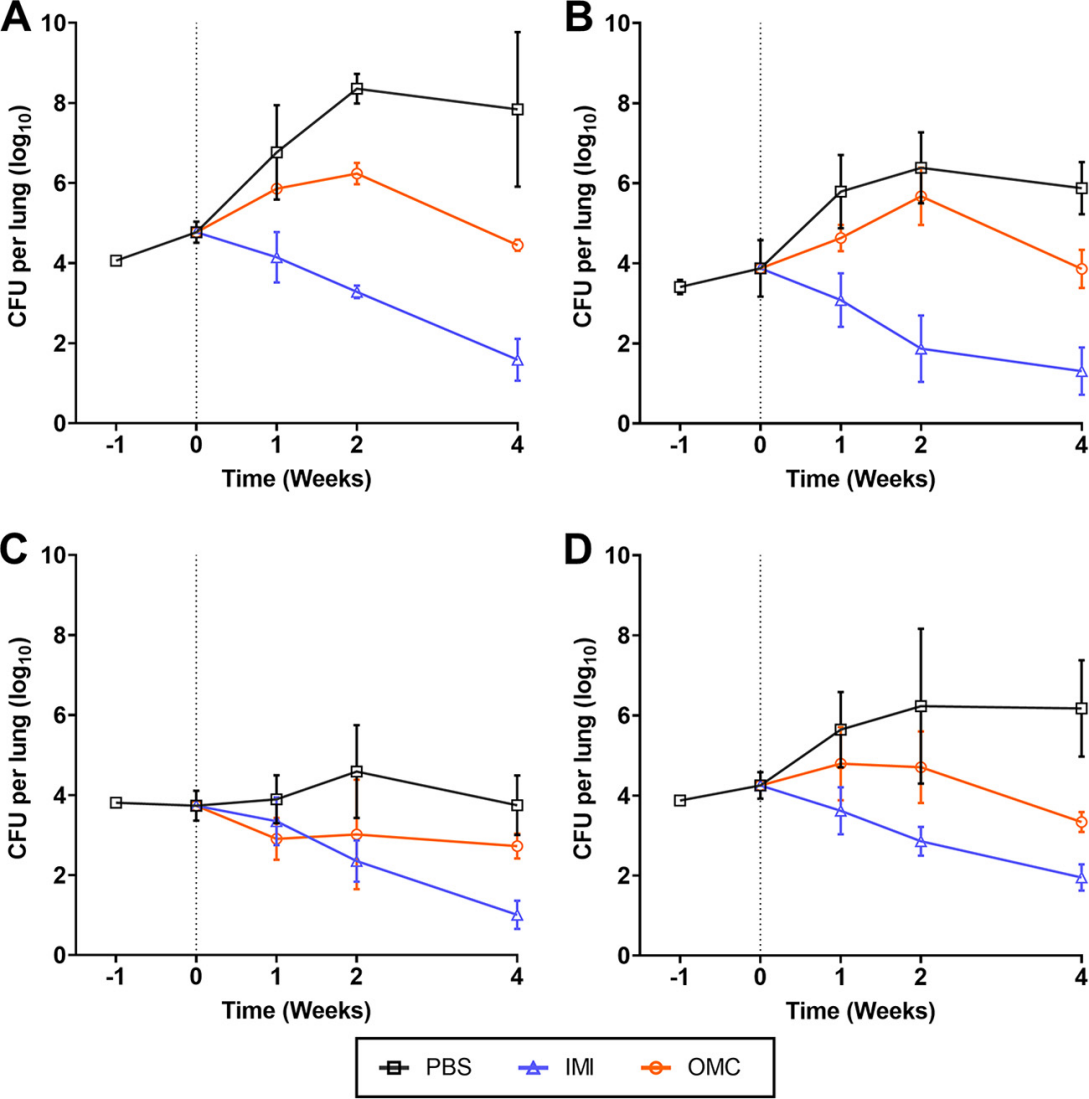
M. abscessus burden in the lungs of mice. C3HeB/FeJ mice were used. All mice were immunosuppressed with dexamethasone. Lung M. abscessus burden assessments at weeks −1, 0, +1, +2, and +4 (*n* = 5 per group per time point; represented as mean ± SD) are shown. Week −1 represents the day after mice were infected with M. abscessus, and week 0 represents the day of antibiotic treatment initiation (denoted with vertical dotted line). Data correspond to mice infected with reference strain ATCC 19977 (A), and clinical isolates M9501 (B), M9529 (C), and M9530 (D). PBS, phosphate-buffered saline control; IMI, imipenem; OMC, omadacycline. PBS and OMC (15 mg/kg) were administered once daily, and IMI (200 mg/kg) was administered twice daily. All agents were administered by subcutaneous injection into the dorsal flank.

In the negative-control group, mice that received 1× phosphate-buffered saline (PBS) treatment at the same frequency as the test group, M. abscessus lung burden increased through the course of infection. In the positive-control group, which received imipenem treatment, M. abscessus lung burden for all four isolates decreased gradually over the course of the study. Omadacycline produced a biphasic effect on lung M. abscessus burden (Fig. 5). At the final time point of 4 weeks of treatment, the lungs of mice harbored >3 log_10_ fewer CFU of ATCC 19977 compared to those of mice in the untreated group. Similarly, omadacycline reduced the lung burdens of M9501, M9529, and M9530 by an average of 2.02 log_10_, 1.02 log_10_, and 2.84 log_10_, respectively, at the conclusion of 4 weeks of treatment. During the first 2 weeks of treatment, the relative growth rate of each M. abscessus isolate was lower than the growth rate in untreated mice. After 2 weeks, omadacycline reduced M. abscessus lung burden and thereby exhibited bactericidal activity. This trend was observed against all isolates except M9529. In M9529-infected mice, omadacycline produced a low-grade reduction in lung burden that was maintained throughout the 4-week treatment.

### MICs of omadacycline against M. abscessus remained unchanged after 4 weeks of exposure in mice.

To assess if prolonged exposure to omadacycline as a monotherapy in this model alters its MIC against M. abscessus, the MICs against ATCC 19977 and M9501 recovered from lung homogenates of mice after 4 weeks of omadacycline treatment were determined along with those against the parent strains. Two colonies from each mouse, five mice per infection group, were randomly selected from 7H11 agar plates inoculated with lung homogenates. Overall, the MICs of omadacycline against the recovered isolates were identical or similar to the MIC value obtained against the parent strains (Table S5). Among the 10 colonies derived from ATCC 19977-infected mice, the MIC of omadacycline remained unchanged for eight isolates (MIC ≤ 0.5 µg/mL). For two isolates, the MIC was 0.75 µg/mL, which is within one dilution. The MICs of omadacycline against all 10 colonies derived from M9501-infected mice were identical to the MIC of omadacycline against the parent M9501 strain.

## DISCUSSION

New antibiotics are needed for the treatment of M. abscessus disease, as demonstrated by the intrinsic resistance of this species to most antibiotics (2–4), the treatment duration of at least 12 months with multidrug regimens containing poorly tolerated agents, and cure rates as low as 25 to 40% (11, 12). There have been no antibiotics evaluated in randomized clinical trials for the treatment of M. abscessus disease, and thus there are no antibiotics approved by the FDA for this indication; current treatment recommendations are based on limited observational studies and consensus from experts. As contemporary M. abscessus isolates exhibit resistance to an increasing number of antibiotics (3, 17), there is emerging interest in repurposing existing antibiotics and developing new ones. Tigecycline, a tetracycline class antibiotic, is among the antibiotics included in the current recommendations and has been valuable in treating this disease (6–8, 10). A study evaluated the utility of tigecycline as part of a multidrug regimen in a cohort of 52 patients, the majority of whom had M. abscessus infection. While the addition of tigecycline produced improvement in 60% of the patients, adverse effects, including nausea and vomiting, were reported in >90% of the cases (58). In the past few years, *in vitro* studies of omadacycline have reported low MICs against M. abscessus and therefore offer promising potential against this bacterium (21–23, 25). Additionally, recent real-world case reports and case series have reported promising clinical outcomes with regimens containing omadacycline in treating M. abscessus lung disease (27, 59–61). These observations have warranted evaluation in a preclinical model that permits efficacy determination of omadacycline alone against multiple M. abscessus isolates in a controlled laboratory setting. This critical knowledge would provide insight and aid in clinical trial design to evaluate omadacycline against M. abscessus disease in patients. Thus, the study described here includes *in vitro* assessment and preclinical efficacy evaluations in a mouse model of pulmonary M. abscessus disease to fill this knowledge gap.

The clinical isolates of M. abscessus included in this study trace their origin to pulmonary infections in various structural lung diseases such as bronchiectasis, cystic fibrosis, and chronic obstructive pulmonary disease and were obtained within the past 15 years (17). In addition, two isolates from a gastrointestinal infection (46) were evaluated in MIC determination studies. We included isolates from M. abscessus subsp. *abscessus* and M. abscessus subsp. *massiliense*, which represent the most frequently isolated subspecies in the clinic. As the omadacycline MIC_90_ of 0.5 µg/mL observed in our study is similar to the MIC_90_ values of omadacycline observed against other Gram-positive species (e.g., Staphylococcus aureus), it is possible that the omadacycline dose currently approved for treatment of community-acquired bacterial pneumonia (CABP) and acute bacterial skin and skin structure infections (ABSSSI) may also prove favorable for the treatment of M. abscessus infections. Indeed, the FDA-approved 300-mg oral dose is currently being evaluated in a phase 2, double-blind, randomized, parallel-group, placebo-controlled study to evaluate the efficacy, safety, and tolerability of oral omadacycline in adults with NTM pulmonary disease caused by M. abscessus complex (ClinicalTrials.gov identifier NCT04922554). Importantly, we demonstrated that mice treated with 15 mg/kg, a dose that best represents the human equivalent exposure of the 300-mg oral dose, is efficacious in a murine model of pulmonary infection due to M. abscessus (20). Reduction in the CFU counts of ATCC 19977, M9501, and M9530 in the lungs of mice occurred after 2 weeks of treatment with omadacycline. During the first 2 weeks, lung burden of these isolates increased, although the CFU counts were consistently lower in mice that received omadacycline than those in the control group that received PBS. Omadacycline is a bacteriostatic agent, which may explain in part the delayed killing that was observed. In addition, changes in pharmacokinetics of omadacycline during the course of infection or alterations in the microenvironment where M. abscessus exist during the infection may also explain the biphasic activity of omadacycline. Additional mechanisms, including changes in metabolism of M. abscessus or the host that affect omadacycline activity, cannot be ruled out. Determining the basis for the delayed bactericidal activity of omadacycline observed against these isolates will require further study.

Additionally, continuous exposure to omadacycline for 4 weeks in mice did not alter the MICs of omadacycline against ATCC 19977 and M9501. A potential limitation of this assessment is that MICs of omadacycline were determined for only 10 colonies originating from ATCC 19977 and M9501. Although these colonies were randomly picked and therefore can be expected to represent the M. abscessus population in mice following continuous exposure to omadacycline for 4 weeks, it would require determination of MIC of omadacycline against all surviving colonies to definitively ascertain if any changes to the MIC occurred. Since we did not observe any alterations in MICs against these two strains, we considered it incremental and potentially unnecessary to repeat this assessment in colonies isolated from mice infected with M9529 and M9530.

In the time-kill assay, concentration- and time-dependent activity of omadacycline was consistently observed against all five M. abscessus isolates tested. The reduction in M. abscessus CFU at 24-h postexposure and subsequent increase in CFU after 24 h at concentrations up to 2× MIC of omadacycline indicates a likely reduction in effective concentration of active omadacycline and hence suggests that continued exposure to the active antibiotic is vital in realizing its anti-M. abscessus activity. This hypothesis is supported by a previous report that demonstrated concentration-dependent killing of M. abscessus by omadacycline over 7 days with the resupplementation of 20% omadacycline to cultures daily (24). Another report noted the preference of utilizing oxyrase in *in vitro* studies to stabilize omadacycline against potential degradation over time (62). A low inoculum of M. abscessus was used in the time-kill assays to minimize introduction of any preexisting spontaneous resistant mutants whose outgrowth would confound the interpretation of CFU levels.

Omadacycline and 17 additional standard of care antibiotics were tested in two biological replicate MIC assays against the 32 M. abscessus isolates, each using two different medium types, CAMHB (Table 1) and Middlebrook 7H9 broth (see Table S1 in the supplemental material). A surprising and clinically relevant observation was that the MIC values obtained for omadacycline and several other antibiotics trended lower in CAMHB compared to those in Middlebrook 7H9 broth but that the MIC values of imipenem trended higher in CAMHB compared to those in Middlebrook 7H9 broth. These results suggest that the selection of an appropriate medium is critical for accurate susceptibility testing and MIC reporting of M. abscessus and that CLSI guidelines should be followed when available. In particular, we recommend that CLSI susceptibility testing guidelines are followed for evaluation of omadacycline MICs against NTM.

Additionally, the MIC of imipenem against M9529 is 256 µg/mL in both CAMHB and Middlebrook 7H9 broth, and it is considered resistant according to CLSI breakpoint guidelines (49) (Table S2) but imipenem was efficacious against this strain in mice (Fig. 5). These observations bring into question whether *in vitro* MICs of imipenem against some M. abscessus isolates in either of these two broths can be informative in making clinical decisions on considering imipenem in treatment regimens. Compared to that of ATCC 19977, M9501, and M9530, growth of M9529 in the lungs of mice was attenuated through the 4-week period (Fig. 5C). M. abscessus isolates exhibit significant heterogeneity in their growth *in vivo*, and more than 3 log_10_ CFU difference in the lungs of C3HeB/FeJ mice between the fastest and slowest growing isolates have been described (35).

Treatment regimens for M. abscessus pulmonary disease require the use of multiple antimicrobials in combination (6–8, 10), and therefore it is critical to confirm that there is absence of antagonistic activity when antimicrobials are combined. No antagonism was observed when omadacycline was combined with any of the antibiotics assessed against any strains in this study. Instead, omadacycline exhibited synergy in combination with several other antibiotics. For example, omadacycline in combination with clarithromycin exhibited synergy against the largest number (54.5%; 6 of 11) of M. abscessus isolates. Because of the small sample size (*n* = 11) and the inclusion of isolates that are resistant to several antibiotics, the actual proportion of clinical isolates against which the combination of omadacycline and clarithromycin may exhibit synergy may be slightly different. Additionally, although omadacycline in combination with rifabutin exhibited synergy against fewer isolates than in combination with clarithromycin, the FICI range of omadacycline plus rifabutin was the narrowest at 0.68 ± 0.20 (mean ± standard deviation [SD]). The FICI values for omadacycline plus clarithromycin showed a slightly wider range, with a mean ± standard deviation of 0.55 ± 0.30. An important finding was that antibiotics with which omadacycline exhibited synergy, including clarithromycin, azithromycin, cefdinir, linezolid, and rifabutin, are available in oral formulation. Therefore, there is potential, pending further evaluation in animal models of efficacy and clinical evaluation in humans, that a fully oral regimen containing omadacycline may be feasible. This type of regimen would reduce the logistical challenges associated with administering prolonged intravenous therapy in an outpatient setting, as is often required for treatment of M. abscessus lung disease.

The most recently published clinical practice guidelines for the treatment of NTM pulmonary disease were published in 2020 but failed to incorporate new antibiotics into these guidelines (10). A subsequent publication in July 2021 by two guideline authors provides updated treatment recommendations for M. abscessus pulmonary infections (63). The authors recommend oral omadacycline as a preferred drug but note that while there is “impressive *in vitro* activity” and reports of “anecdotal clinical successes,” it is not yet clear if treatment with omadacycline will contribute to better outcomes compared to those under the current regimens (63). In addition, the FDA granted omadacycline orphan drug designation in August 2021 for the treatment of infections caused by NTM, and this designation includes NTM pulmonary disease caused by the M. abscessus complex, the focus of the ongoing phase 2 clinical trial (ClinicalTrials.gov identifier NCT04922554). In total, the data presented in our study provide additional support to this new treatment recommendation, as well as to the continued study of omadacycline in patients with pulmonary disease due to M. abscessus.

## MATERIALS AND METHODS

### Ethics.

Animal procedures used in the studies described here were performed in adherence to the Johns Hopkins University Animal Care and Use Committee and to the national guidelines.

### Bacterial strains and *in vitro* growth conditions.

M. abscessus reference strain ATCC 19977 (47) was purchased from ATCC (Manassas, VA) and authenticated by sequencing its genome (48). Strains M9563 and M9535, isolated from gastrointestinal infections, were kind gifts from Thomas Byrd, University of New Mexico School of Medicine, and correspond to strains 390R and 390V, respectively, as previously described (46, 64). The remainder of the isolates were obtained from Nicole Parrish at the Johns Hopkins University Clinical Microbiology Laboratory, who isolated them from cystic fibrosis and bronchiectasis patients between 2006 and 2018 (17). All isolates were grown in either cation adjusted Mueller-Hinton Broth (CAMHB) (catalog no. 90922; Sigma-Aldrich) or Middlebrook 7H9 broth (catalog no. 271310; Difco), as specified. Middlebrook 7H9 broth was supplemented with 0.5% glycerol and 10% albumin-dextrose-catalase enrichment. M. abscessus cultures were grown in an orbital shaker at 220 rpm and 37°C. Omadacycline was obtained from Paratek Pharmaceuticals, Inc. All antibiotics were purchased from Sigma-Aldrich, with the exception of bedaquiline (catalog no. A193466; Ambeed) and imipenem (Octagon Chemicals Limited). Mouse lung homogenates were cultured on Middlebrook 7H11 selective agar (catalog no. 283810; Difco) supplemented with 10% albumin-dextrose-catalase enrichment, 50 µg/mL carbenicillin (catalog no. C46000; Research Products International), and 50 µg/mL cycloheximide (catalog no. C7698; Sigma-Aldrich) as described previously (65).

### Determination of MICs.

The standard broth microdilution method (66, 67) with conditions specified in the CLSI guidelines specific for M. abscessus (49) was used to determine the MIC of each antibiotic against 32 different M. abscessus isolates (Table 1). Sterile deionized water was used to dissolve powdered drug stocks; if insoluble in water, they were dissolved in dimethyl sulfoxide (DMSO) at a high concentration and then diluted in water. CAMHB broth was used as specified in the CLSI guidelines (49), and Middlebrook 7H9 broth was also used separately. Twofold serial dilutions of each antibiotic were prepared in each broth, generating final antibiotic concentrations ranging from 256 µg/mL to 0.06 µg/mL in 200 µl final volume in each well of a 96-well culture plate. Using an exponentially growing culture of each isolate, 10^5^ CFU of M. abscessus was inoculated into each well. As positive and negative controls, two wells containing 10^5^ CFU of M. abscessus without drug and two wells containing broth alone were included in each plate. Plates were incubated at 30°C for 72 h in accordance with CLSI guidelines. A Sensititre manual viewbox was used to determine the growth or lack thereof of M. abscessus, and the lowest concentration at which M. abscessus growth was not observed was recorded as the MIC of the antibiotic. Each MIC assay was performed in duplicate, and the final MIC reported in Table 1 was an average of the biological replicates of the assays.

### Checkerboard titration assay.

This assay, a modification of the standard broth microdilution assay, was performed as described previously (50, 68). Briefly, in sterile U-bottomed 96-well plates with 300-µl well capacity, stock solutions of two antibiotics were added to CAMHB broth, each starting at 2× MIC and serially diluted up to 1/64× MIC, such that all possible 2-fold dilution combinations from 2× to 1/64× MIC of each antibiotic were included. Using an exponentially growing culture of each strain, 10^5^ CFU of M. abscessus was inoculated into each well, and positive and negative controls were included as described above. In accordance with CLSI guidelines, plates were incubated for 72 h at 30°C and evaluated for M. abscessus growth using a Sensititre manual viewbox. The fractional inhibitory concentration index (FICI) was calculated as described previously (50). Per the stringent interpretation recommended, an FICI of ≤0.5 was interpreted as synergy, an FICI of >0.5 to 4 as indifference, and an FICI of >4 as antagonism (51). The reference strain ATCC 19977 and 10 clinical isolates, M9501, M9502, M9507, M9510, M9513, M9522, M9524, M9526, M9529, and M9530, were included. Omadacycline in combination with 14 antibiotics were assayed. A total of 165 separate assays testing 14 antibiotic combinations against 11 strains were performed. For those combinations that produced a FICI of ≤0.5, a biological repeat of the assay was performed to verify synergy.

### Time-kill assay.

Activities of omadacycline alone and in combination with clarithromycin, azithromycin, cefdinir, linezolid, and rifabutin against the reference strain ATCC 19977 and four randomly selected clinical isolates, M9510, M9526, M9529, and M9530, were determined. Each M. abscessus isolate was grown in CAMHB broth to the exponential phase, and a suspension at an optical density *A*_600_ of 0.01 was prepared by diluting the culture in fresh broth. Culture tubes (50 mL) containing omadacycline at 0.5×, 1×, 2×, 4×, 8×, and 16× MIC specific to each isolate in 4.8 mL CAMHB broth were prepared and inoculated with 200 µl of the M. abscessus suspension, or ∼10^5^ CFU. A positive control for growth of M. abscessus without omadacycline was included in each assessment. The samples were incubated in an orbital shaker at 220 rpm and 37°C. At 0, 1, 3, and 7 days, a 100-µl aliquot was obtained from each sample, and appropriate 10-fold serial dilutions were prepared in CAMHB broth, inoculated onto CAMHB agar, and CFU enumerated after 3 days of incubation at 37°C. Similarly, to determine the time-kill activities of omadacycline in combination with another antibiotic, the five M. abscessus isolates were exposed to omadacycline and clarithromycin, azithromycin, cefdinir, linezolid, or rifabutin at 1×, 0.5×, and 0.25× MIC of each antibiotic specific to each strain, grown, and CFU determined. For each sample, CFU counts were converted to CFU/mL, and mean ± standard deviation data versus time were plotted.

### Determination of omadacycline PK parameters in C3HeB/FeJ mice and in BALB/c mice.

Uninfected C3HeB/FeJ mice (6 to 8 weeks old, female) were injected subcutaneously with 7.5, 15, or 30 mg/kg omadacycline solution in 1× PBS. Blood samples were taken via terminal cardiac puncture at 0.5, 1, 2, 3, 6, 12, and 24 h postinjection, and at each time point, a single blood sample was collected from a single mouse. Five mice per dose and time point were utilized. Uninfected BALB/c mice (6 to 8 weeks old, female) were injected intraperitoneally with 2.5, 7.5, 15, or 30 mg/kg omadacycline, and blood samples were taken via terminal cardiac puncture at 0.25, 0.5, 1, 2, 6, 12, and 24 h postinjection. Blood was transferred to lithium heparin vials and centrifuged at 1,500 × *g* for 10 min to separate the plasma. Plasma was stored at −80°C until omadacycline concentration analysis.

The mean (±SD) plasma concentration versus scheduled time points for uninfected BALB/c mice are shown in Fig. 4A and B. These mean concentration profile data were used to estimate the PK parameters of omadacycline in plasma by standard noncompartmental methods using a WinNonlin (Phoenix) validated SAS program for all dose groups. For the calculation of PK parameters, values below the limit of quantification (BLQ) before the first quantifiable concentration were treated as zero. Values BLQ after the first quantifiable concentration were retested and confirmed and were included in the analyses. Missing concentrations were treated as missing.

To determine omadacycline concentrations in M. abscessus-infected C3HeB/FeJ mice, mice that had been infected were treated with 15 mg/kg omadacycline as described, and blood samples were taken via cardiac puncture from 5 mice at 24 h posttreatment at 0, 1, 2, and 4 weeks postinfection. Blood was transferred to lithium heparin vials and centrifuged at 1,500 × *g* for 10 min to separate the plasma. Plasma was stored at −80°C until omadacycline concentration analysis.

### Determination of omadacycline concentrations.

Omadacycline plasma concentrations were determined by the Institute for Clinical Pharmacodynamics (ICPD; Schenectady, NY) using a qualified liquid chromatography-tandem mass spectrometry (LC-MS/MS) method. Samples were subjected to protein precipitation and chromatographically separated on an Ace 3 C_18_ high-performance liquid chromatography (HPLC) column (100 mm × 3 mm, 3-µm particle size; Advanced Chromatography Technologies, Ltd.) using a Sciex Exion LC AC system. Analyte molecules were detected using a Sciex 5500 mass spectrometer scanning in positive ion mode. Omadacycline tosylate drug substance (omadacycline) was used to prepare the stock standard and working standard omadacycline solutions. d9-Omadacycline was used as the internal standard for the analysis of omadacycline. The peak areas of omadacycline and its internal standard, d9-omadacycline, were acquired using Analyst 1.6.3 software (Sciex, Framingham, MA). The calibration curves were obtained by fitting the peak area ratios of omadacycline/d9-omadacycline and the standard concentrations to a linear 1/×2 regression model using Analyst 1.6.3 software. The equations of the calibration curves were then used to interpolate the concentrations of omadacycline in the samples using their peak area ratios. The peak areas and peak area ratios used for the calculations were rounded to three precision points.

### Mice, infection, and efficacy studies.

C3HeB/FeJ mice (female, 5 to 6 weeks old) were procured from Jackson Laboratories (Bar Harbor, ME). As described in the protocol for a mouse model of pulmonary M. abscessus infection (35), mice were treated daily with 5 mg/kg/day dexamethasone beginning 1 week prior to infection with M. abscessus and continuing throughout the duration of the study. M. abscessus strains ATCC 19977, M9501, M9529, and M9530 were used to infect mice. Infection with each strain was performed separately; 110 mice were infected with each strain. In a Glas-Col inhalation exposure system, all 110 mice were infected concurrently with aerosol generated from 10 mL of exponentially growing M. abscessus culture diluted to an *A*_600_ of 0.1 in sterile 1× PBS (pH 7.4) according to the manufacturer’s instructions (Glas-Col, Terre Haute, Indiana). The infection cycle included preheating for 15 min, aerosol nebulization for 30 min, and cloud decay for 30 min, followed by surface decontamination for 15 min. This study was conducted in two phases. The first phase included evaluation of omadacycline efficacy in mice infected with ATCC 19977 and M9501. At the conclusion of this phase, two M. abscessus colonies were randomly selected from 7H11 agar plates containing lung homogenates of each mouse following completion of 4 weeks of daily omadacycline treatment. The MIC of omadacycline was determined against these 20 colonies to assess if daily exposure to omadacycline for 4 weeks altered its MIC against these strains. In the second phase, efficacy of omadacycline was evaluated in mice infected with M9529 and M9530. An identical protocol was used in both phases, but the studies were separated in time. Five mice per infecting strain group were sacrificed at 24 h postinfection (designated week −1), and lungs were homogenized, inoculated onto Middlebrook 7H11 selective plates described above, incubated at 37°C for 5 days, and CFU recorded to determine the initial M. abscessus burden in the lungs of mice. Similarly, five mice were sacrificed at 1 week postinfection (week zero) and CFU enumerated. CFU counts from each mouse lung were converted into CFU per lung, comprising the average of three consecutive steps of a 10-fold dilution series of a given lung sample. Mean CFU ± standard deviation of lung M. abscessus burden in five mice per group per time point was plotted determine the growth of each isolate under conditions tested.

### Antibiotic regimens.

At 1 week postinfection (week zero), mice infected with each strain were further divided into three groups of 15 mice per group. All antibiotics and control treatments were administered via subcutaneous injection in the dorsal flank of hind limbs. Mice in the negative-control group were treated once daily with 200 µl sterile 1× PBS. Mice in the positive-control group were administered imipenem, and mice in the test group were administered omadacycline. Powdered omadacycline and imipenem were resuspended in sterile 1× PBS (pH 7.4; Quality Biologicals) for administration of 15 mg/kg/every 25 h (q24h) and 200 mg/kg/q12h of omadacycline and imipenem, respectively, using a 200-µl bolus. An average body weight of 25 g per mouse was considered based on our past experience with this mouse strain, age, and sex (35). Therefore, omadacycline and imipenem were prepared at concentrations of 1.875 mg/mL and 25 mg/mL for administration. Omadacycline dissolved completely in 1× PBS (pH 7.4) and produced a solution, so a single batch of omadacycline was produced for the experiment, and aliquots for each day of administration were stored at −20°C and thawed at the time of administration. Imipenem dissolved incompletely in PBS and produced a fine suspension. Imipenem powder for each administration was weighed, stored at −20°C, and freshly resuspended in 1× PBS (pH 7.4) moments prior to administration. All treatments were administered via subcutaneous injection into the dorsal flank using a syringe with a 27-gauge needle. Daily is defined as 7 days a week. At 1 week (week +1), 2 weeks (week +2), and 4 weeks (week +4) from the time of antibiotic treatment initiation, five mice per treatment arm were sacrificed, and M. abscessus lung burden was determined as described above.

### Data analysis.

CFU data from *in vitro* and *in vivo* studies were analyzed to determine mean plus or minus standard deviation for each time point in each experimental group and graphed using GraphPad Prism v8.4.3.
