# Supplementary material for: Potency of Omadacycline against Mycobacteroides abscessus Clinical Isolates In Vitro and in a Mouse Model of Pulmonary Infection
Source: Antimicrob Agents Chemother. 2022 Jan 18;66(1):e01704-21. doi: 10.1128/AAC.01704-21 (PMC8765394; doi:10.1128/AAC.01704-21)
Supplement: Supplemental file 1 — Supplemental figures and tables. Download AAC.01704-21-s0001.pdf, PDF file, 0.9 MB [file aac.01704-21-s0001.pdf]

SUPPLEMENTARY INFORMATION

SUPPLEMENTARY FIGURES AND TABLES

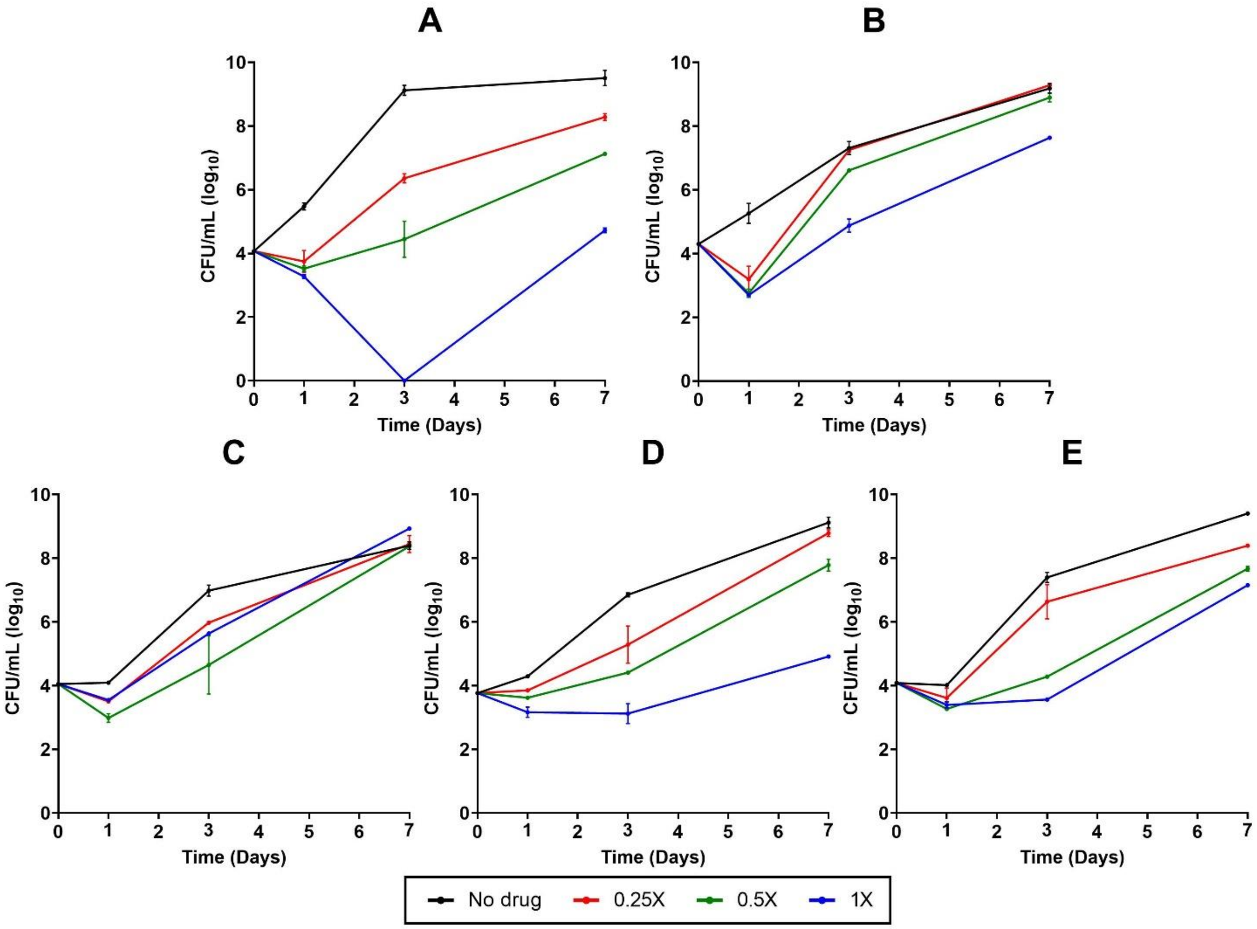

Supplementary Figure 1: Time-kill activity of omadacycline and clarithromycin against five *M. abscessus* isolates*. M. abscessus* strains (A) ATCC 19977, and clinical isolates (B) M9510, (C) M9526, (D) M9529, and (E) M9530 were exposed to a combination of omadacycline and clarithromycin at 1X, 0.5X and 0.25X MIC of each drug specific to each strain and no drug in CAMHB broth and surviving colonies were recovered in duplicate on CAMHB agar at 1, 3 and 7 days and enumerated (mean±SD).

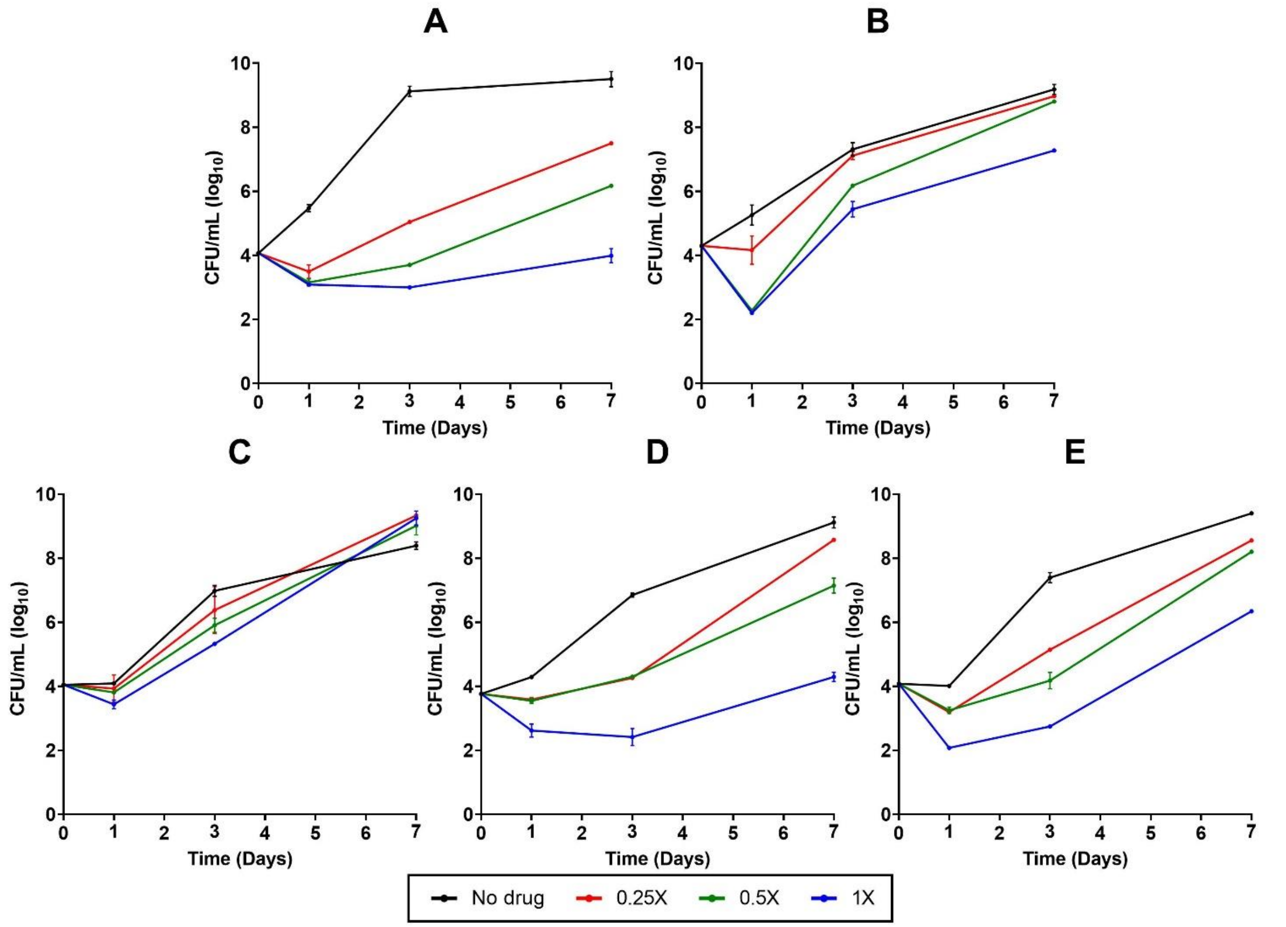

Supplementary Figure 2: Time-kill activity of omadacycline and azithromycin against five *M. abscessus* isolates*. M. abscessus* strains (A) ATCC 19977, and clinical isolates (B) M9510, (C) M9526, (D) M9529, and (E) M9530 were exposed to a combination of omadacycline and azithromycin at 1X, 0.5X and 0.25X MIC of each drug specific to each strain and no drug in CAMHB broth and surviving colonies were recovered in duplicate on CAMHB agar at 1, 3 and 7 days and enumerated (mean±SD).

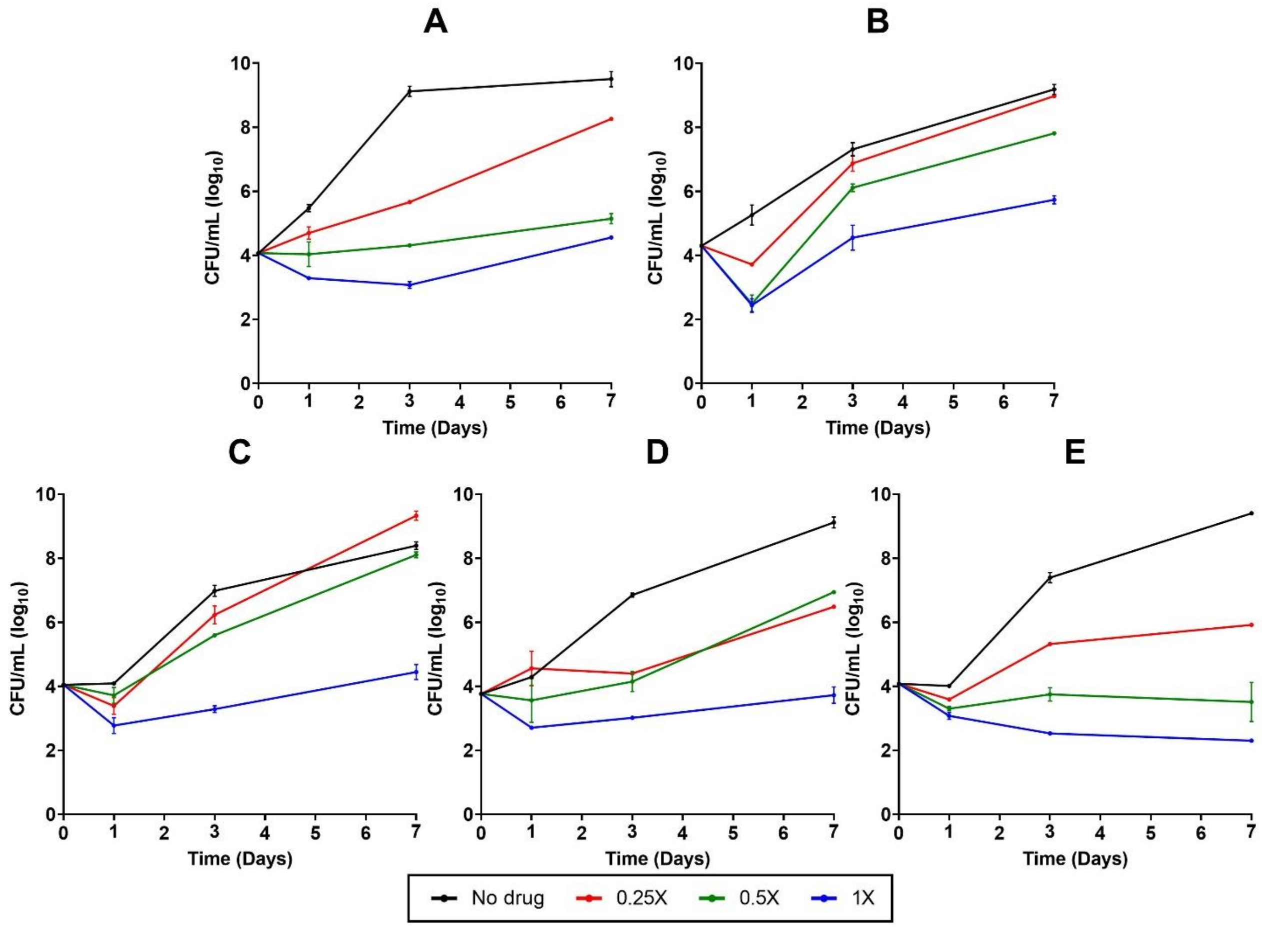

Supplementary Figure 3: Time-kill activity of omadacycline and cefdinir against five *M. abscessus* isolates*. M. abscessus* strains (A) ATCC 19977, and clinical isolates (B) M9510, (C) M9526, (D) M9529, and (E) M9530 were exposed to a combination of omadacycline and cefdinir at 1X, 0.5X and 0.25X MIC of each drug specific to each strain and no drug in CAMHB broth and surviving colonies were recovered in duplicate on CAMHB agar at 1, 3 and 7 days and enumerated (mean±SD).

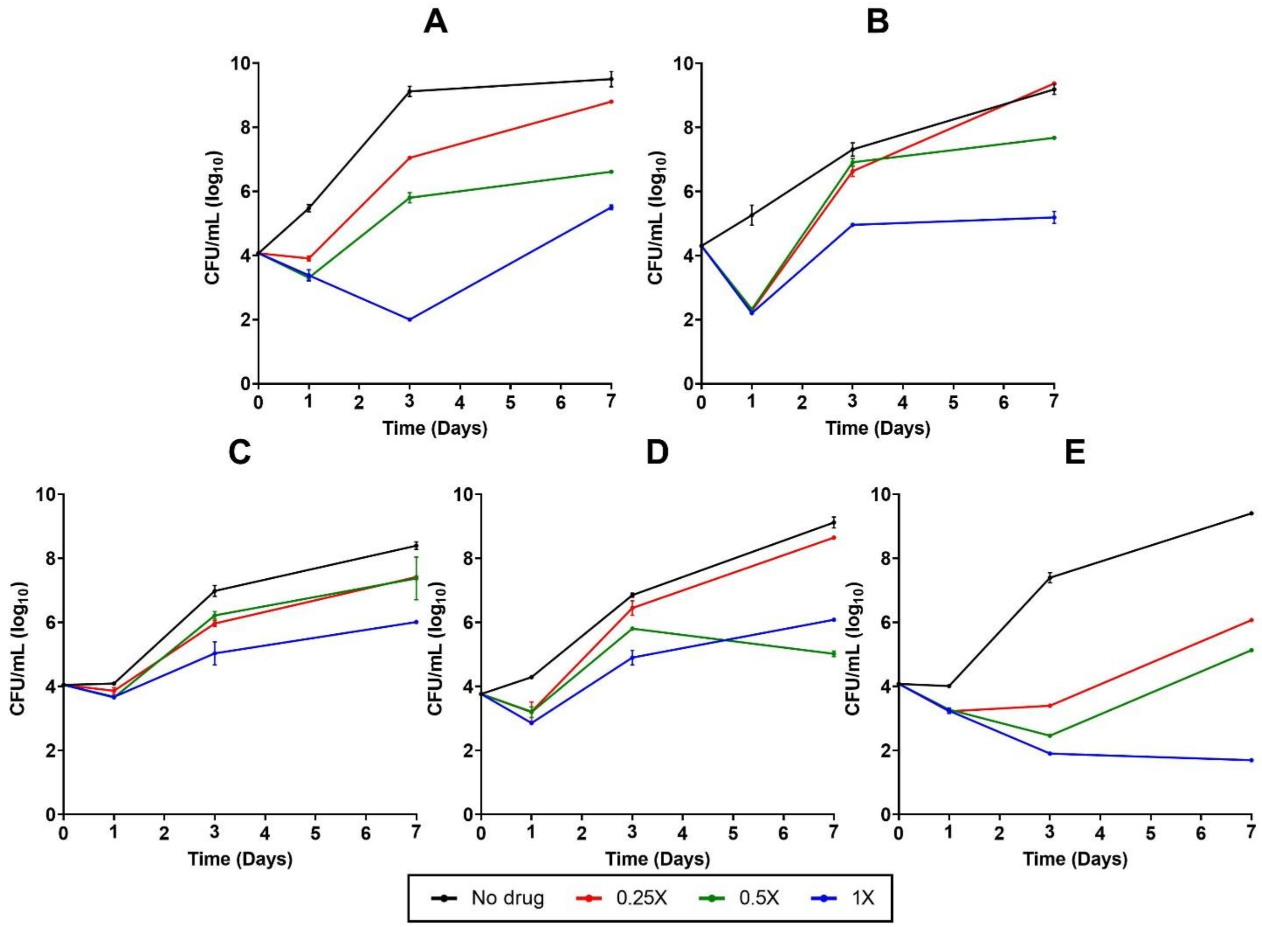

Supplementary Figure 4: Time-kill activity of omadacycline and linezolid against five *M. abscessus* isolates*. M. abscessus* strains (A) ATCC 19977, and clinical isolates (B) M9510, (C) M9526, (D) M9529, and (E) M9530 were exposed to a combination of omadacycline and linezolid at 1X, 0.5X and 0.25X MIC of each drug specific to each strain and no drug in CAMHB broth and surviving colonies were recovered in duplicate on CAMHB agar at 1, 3 and 7 days and enumerated (mean±SD).

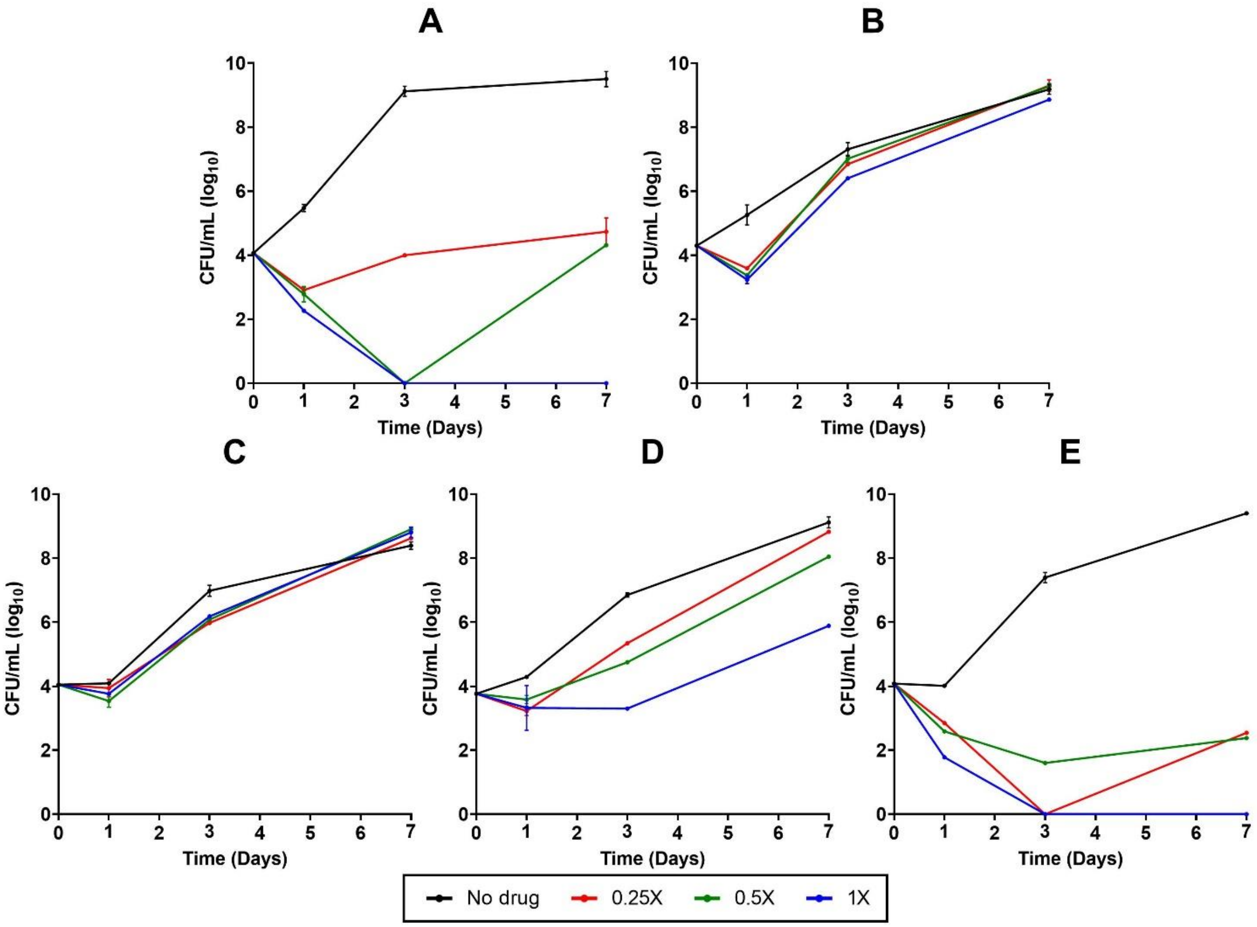

Supplementary Figure 5: Time-kill activity of omadacycline and amikacin against five *M. abscessus* isolates*. M. abscessus* strains (A) ATCC 19977, and clinical isolates (B) M9510, (C) M9526, (D) M9529, and (E) M9530 were exposed to a combination of omadacycline and amikacin at 1X, 0.5X and 0.25X MIC of each drug specific to each strain and no drug in CAMHB broth and surviving colonies were recovered in duplicate on CAMHB agar at 1, 3 and 7 days and enumerated (mean±SD).

Supplementary Table 1: MIC (µg/ml) of omadacycline and select antibiotics against 31 *M. abscessus* clinical isolates and reference strain ATCC 19977 in Middlebrook 7H9 broth.

**Subspecies**	**Isolate ID**	**OMC**	**TGC**	**AMK**	**CLR**	**AZM**	**IMI**	**DOR**	**FOX**	**CDR**	**CAZ**	**AMX**	**VAN**	**TEC**	**LZD**	**CFZ**	**MOX**	**RFB**	**BDQ**
abscessus	**19977**	0.5	0.125	16	8	32	8	16	64	128	>256	>256	>256	>256	64	2	16	16	<0.06
abscessus	**M9501**	0.75	0.188	24	2	32	4	12	64	96	>256	>256	>256	>256	96	2	64	16	<0.06
abscessus	**M9503**	0.5	0.125	24	32	128	6	12	64	64	>256	>256	>256	>256	128	2	32	16	<0.06
abscessus	**M9507**	0.25	0.125	>256	32	128	24	64	64	128	>256	>256	>256	>256	128	2	64	16	<0.06
abscessus	**M9513**	0.5	0.375	16	8	64	8	48	32	64	>256	>256	>256	>256	64	2	24	32	<0.06
abscessus	**M9522**	0.5	0.125	12	16	64	8	12	24	96	>256	>256	>256	>256	64	2	16	24	<0.06
abscessus	**M9525**	0.375	0.188	>256	32	128	32	48	64	192	>256	>256	>256	>256	64	2	24	24	<0.06
abscessus	**M9526**	0.5	0.125	>256	32	128	32	32	64	128	>256	>256	>256	>256	64	2	64	32	<0.06
abscessus	**M9527**	0.5	0.188	>256	32	128	32	64	64	128	>256	>256	>256	>256	128	2	64	16	<0.06
abscessus	**M9528**	0.5	0.188	>256	32	128	32	32	64	128	>256	>256	>256	>256	64	2	64	8	<0.06
abscessus	**M9529**	2	0.75	>256	32	128	256	256	32	128	>256	>256	>256	>256	128	2	128	32	<0.06
abscessus	**M9530**	1	0.25	32	64	128	12	8	32	64	>256	>256	>256	>256	96	2	32	48	<0.06
abscessus	**M9531**	1	0.5	32	64	128	32	16	32	128	>256	>256	>256	>256	128	2	32	16	<0.06
massiliense	**M9502**	1	0.25	24	1	32	8	16	32	32	>256	>256	>256	>256	64	2	16	32	<0.06
massiliense	**M9504**	0.5	0.188	24	2	16	8	8	32	64	>256	>256	>256	>256	64	2	8	32	<0.06
massiliense	**M9505**	0.75	0.375	24	1.5	24	8	12	32	64	>256	>256	>256	>256	64	2	24	8	<0.06
massiliense	**M9509**	0.5	0.125	8	0.25	4	8	8	16	16	>256	>256	>256	>256	32	1	8	8	<0.06
massiliense	**M9510**	1.5	1.5	24	1	64	24	64	64	128	>256	>256	>256	>256	128	2	64	16	<0.06
massiliense	**M9514**	0.75	0.375	24	1	32	12	24	48	32	>256	>256	>256	>256	≥256	2	64	16	<0.06
massiliense	**M9515**	1.5	1	24	1.5	32	32	48	128	96	>256	>256	>256	>256	128	2	128	16	<0.06
massiliense	**M9517**	0.75	0.188	24	2	32	8	12	32	128	>256	>256	>256	>256	128	2	12	32	<0.06
massiliense	**M9521**	0.5	0.125	24	1	16	8	8	32	64	>256	>256	>256	>256	64	2	8	16	<0.06
nd	**M9508**	0.5	0.188	32	32	128	12	16	32	128	>256	>256	>256	>256	128	2	16	48	<0.06
nd	**M9518**	0.5	0.375	32	24	64	16	24	32	128	>256	>256	>256	>256	96	3	24	24	<0.06
nd	**M9519**	1	0.75	16	32	128	8	12	32	128	>256	>256	>256	>256	128	2	16	32	<0.06
nd	**M9523**	0.25	0.125	32	1	8	16	16	64	128	>256	>256	>256	>256	128	2	12	32	<0.06
nd	**M9524**	1	0.375	24	2	24	8	12	48	64	>256	>256	>256	>256	64	2	8	32	<0.06
nd	**M9533**	1	0.125	32	32	128	32	12	24	128	>256	>256	>256	>256	128	2	16	12	<0.06
nd	**M9534**	0.75	0.188	32	2	32	12	16	32	128	>256	>256	>256	>256	128	2	16	16	<0.06
nd	**M9551**	1	0.25	64	2	32	24	32	64	128	>256	>256	>256	>256	128	2	32	16	<0.06
nd	**M9563**	1	0.5	32	32	128	24	24	64	128	>256	>256	>256	>256	128	2	32	32	<0.06
nd	**M9565**	0.375	0.188	24	4	32	12	12	32	64	>256	>256	>256	>256	48	2	8	8	<0.06
**Range**		0.25-2	0.125-1.5	8->256	0.25-64	4-128	4-256	8-256	16-128	16-192	>256	>256	>256	>256	32->256	1-3	8-128	8-48	<0.06
**MIC_{50}**		0.5	0.188	24	8	64	12	16	32	128	>256	>256	>256	>256	96	2	24	16	<0.06
**MIC_{90}**		1	0.75	>256	32	128	32	64	64	128	>256	>256	>256	>256	128	2	64	32	<0.06

MIC values are average of two biological replicates. OMC, omadacycline; TGC, tigecycline; AMK, amikacin; CLR, clarithromycin; AZM, azithromycin; IMI, imipenem; DOR, doripenem; FOX, cefoxitin; CDR, cefdinir; CAZ, ceftazidime; AMX, amoxicillin; VAN, vancomycin; TEC, teicoplanin; LZD, linezolid; CFZ, clofazimine; MOX, moxifloxacin; RFB, rifabutin, and BDQ, bedaquiline. '*nd*' denotes *M. abscessus* isolates whose subspecies has not been determined.

Supplementary Table 2: Antimycobacterial Agents and Breakpoints for Rapidly Growing Mycobacteria.

Antimicrobial Agent	MIC (µg/ml)		
	S	I	R
Amikacin (IV)	≤16	32	≥64
Cefoxitin, Cefdinir	≤16	32-64	≥128
Clarithromycin, Azithromycin	≤2	4	≥8
Imipenem	≤4	8-16	≥32
Linezolid	≤8	16	≥32
Moxifloxacin	≤1	2	≥4

Susceptible (S), intermediate (I), and resistant (R) MIC breakpoints against *M. abscessus* of tested antimicrobial agents in accordance with CLSI M62: Performance Standards for Susceptibility Testing of Mycobacteria, *Nocardia* spp., and Other Aerobic Actinomycetes (1st Edition, 2019).

Supplementary Table 3: Fractional Inhibitory Concentration Index (FICI) and MICs of drugs in combination with Omadacycline tested against *M. abscessus* that demonstrated synergy.

Companion drug	Number of *Mab* stains against which synergy was observed in combination with omadacycline	FICI range	MIC of companion drug when tested alone / MIC of companion drug when combined with omadacycline (µg/ml)					
			Isolate #1	Isolate #2	Isolate #3	Isolate #4	Isolate #5	Isolate #6
Clarithromycin	6 of 11 (55%)	0.17-0.5	4/0.5	0.75/0.13	0.5/0.13	2/0.25	1/0.13	3/0.13
Azithromycin	4 of 11 (36%)	0.25-0.34	24/1	64/1	64/2	4/1		
Cefdinir	4 of 11 (36%)	0.38-0.5	64/8	64/8	32/8	64/16		
Linezolid	4 of 11 (36%)	0.22-0.39	64/8	32/2	64/2	32/4		
Rifabutin	3 of 11 (27%)	0.33-0.49	8/1	16/1	16/2			

FICI ≤ 0.5 indicates synergy. 'Companion drug' refers to the drug that was combined with omadacycline. The 'FICI range' represents the range of FICIs observed against the strains with FICI ≤ 0.5. All MICs that resulted after combination with omadacycline were confirmed susceptible according to CLSI guidelines for the companion drug (Supplementary Table 2) except for rifabutin, where MIC breakpoints against *M. abscessus* are not established.

Supplementary Table 4: Checkerboard assay of omadacycline and clarithromycin against *M. abscessus* isolate M9507.

Plate ID: CLR04				Omadacycline + Clarithromycin					Mab strain: M9507	
↓CLR	OMC→	2.0	1.0	0.5	0.25	0.125	0.063	0.031	0.016	μg/mL	
μg/mL		1	2	3	4	5	6	7	8		10
8.0	A	-	-	-	-	-	-	-	-		+
4.0	B	-	-	-	-	-	-	-	-		+
2.0	C	-	-	-	-	-	-	-	-		+
1.0	D	-	-	-	-	-	-	-	-		+
0.5	E	-	-	-	-	-	-	-	-		-
0.25	F	-	-	-	-	-	+	+	+		-
0.125	G	-	-	-	-	+	+	+	+		-
0.063	H	-	-	-	-	+	+	+	+		-
FICI = 0.33						INTERPRETATION = SYNERGY					

The actual setup of checkerboard assay of omadacycline and clarithromycin in a 96-well microtiter plate and results are shown. The MICs of omadacycline and clarithromycin when tested alone against *M. abscessus* isolate M9507 are 0.5 and 4.0 µg/ml, respectively (Table 1). These MICs are highlighted in red and the row and column in the 96-well plate that correspond to the MICs of clarithromycin and omadacycline are shaded in orange. M9501 inoculated into CAMHB broth but without drug, therefore representing positive control for growth are included in column 10, rows A-D. Wells in this column, rows E-F, contain CAMHB broth only. Growth of M9501 or absence of growth in each well is denoted by '+' and '-', respectively. The wells shaded in green represent concentrations of omadacycline and clarithromycin that are ≤0.25x MIC of either drug, and therefore would denote synergy if growth is not observed. The lowest concentration of clarithromycin at which growth of M9501 could not be observed is 0.5 µg/ml, irrespective of omadacycline concentration. This is the concentration shown in Supplementary Table 3, Row 2, column 4 and represents the MIC of clarithromycin against M9501 when used in combination with omadacycline.

Supplementary Table 5: MICs of omadacycline vs. colonies of *M. abscessus* ATCC 19977 and M9501 recovered from lungs of mice that received four weeks of daily omadacycline treatment.

ID of colonies recovered from ATCC 19977 infected mice	MIC of omadacycline (µg/ml)
ATCC 19977	0.50
2101DN	0.50
2102DN	0.50
2103DN	0.50
2104DN	0.375
2105DN	0.75
2106DN	0.50
2107DN	0.75
2108DN	0.50
2109DN	0.50
2110DN	0.50
**ID of colonies recovered from M9501 infected mice**	
M9501	0.50
2111DN	0.50
2112DN	0.50
2113DN	0.50
2114DN	0.50
2115DN	0.50
2116DN	0.50
2117DN	0.50
2118DN	0.50
2119DN	0.50
2110DN	0.50

MICs (µg/ml) of omadacycline against the strains were determined in CAMHB broth. Average MIC of two biological replicate assays were reported. Two *M. abscessus* colonies were randomly selected from 7H10 agar plates containing lung homogenates of each mouse, five mice per infection group, following completion of 4 weeks of daily omadacycline treatment.
